# Multi‐Arm PEG/Peptidomimetic Conjugate Inhibitors of DR6/APP Interaction Block Hematogenous Tumor Cell Extravasation

**DOI:** 10.1002/advs.202003558

**Published:** 2021-03-18

**Authors:** Liting Wang, Qing Shen, Hongze Liao, Hao Fu, Qi Wang, Jian Yu, Wei Zhang, Chuanrong Chen, Yang Dong, Xupeng Yang, Qianqian Guo, Jiali Zhang, Jian Zhang, Wei Zhang, Houwen Lin, Yourong Duan

**Affiliations:** ^1^ State Key Laboratory of Oncogenes and Related Genes Shanghai Cancer Institute School of Biomedical Engineering Renji Hospital School of Medicine Shanghai Jiao Tong University Shanghai 200032 China; ^2^ Research Center for Marine Drugs State Key Laboratory of Oncogenes and Related Genes Department of Pharmacy Renji Hospital School of Medicine Shanghai Jiao Tong University Shanghai 200127 China; ^3^ Shanghai Key Laboratory of Functional Materials Chemistry School of Chemistry and Molecular Engineering East China University of Science and Technology Shanghai 200237 China; ^4^ Department of Pathophysiology Key Laboratory of Cell Differentiation and Apoptosis of Ministry of Education Shanghai Jiao Tong University School of Medicine Shanghai 200025 China

**Keywords:** anti‐hematogenous metastatic activities, polymer–peptidomimetic conjugates, protein–protein interactions, structure‐based drug design

## Abstract

The binding of amyloid precursor protein (APP) expressed on tumor cells to death receptor 6 (DR6) could initiate the necroptosis pathway, which leads to necroptotic cell death of vascular endothelial cells (ECs) and results in tumor cells (TCs) extravasation and metastasis. This study reports the first inhibitor of DR6/APP interaction as a novel class of anti‐hematogenous metastatic agent. By rationally utilizing three combined strategies including selection based on phage display library, d‐retro‐inverso modification, and multiple conjugation of screened peptidomimetic with 4‐arm PEG, the polymer–peptidomimetic conjugate PEG‐tAHP‐DRI (tetra‐(D‐retro‐inverso isomer of AHP‐12) substitued 4‐arm PEG_5k_) is obtained as the most promising agent with the strongest binding potency (*K*_D_ = 51.12 × 10^−9^ m) and excellent pharmacokinetic properties. Importantly, PEG‐tAHP‐DRI provides efficient protection against TC‐induced ECs necroptosis both in vitro and in vivo. Moreover, this ligand exhibits prominent anti‐hematogenous metastatic activity in serval different metastatic mouse models (B16F10, 4T1, CT26, and spontaneous lung metastasis of 4T1 orthotopic tumor model) and displays no apparent detrimental effects in preliminary safety evaluation. Collectively, this study demonstrates the feasibility of exploiting DR6/APP interaction to regulate hematogenous tumor cells transendothelial migration and provides PEG‐tAHP‐DRI as a novel and promising inhibitor of DR6/APP interaction for developments of anti‐hematogenous metastatic therapies.

## Graphical abstract

Binding of amyloid precursor protein (APP) and death receptor 6 (DR6) is shown to initiate the necroptosis pathway and lead to tumor cells metastasis. The combined strategies of selection based on phage display library, d‐retro‐inverso modification, and multiple conjugation result in the development of conjugate PEG‐tAHP‐DRI (tetra‐(D‐retro‐inverso isomer of AHP‐12) substitued 4‐arm PEG_5k_) that blocks APP‐DR6 interactions and shows promising anti‐hematogenous metastatic activity both in vitro and in vivo.

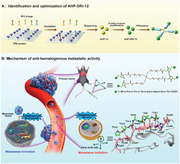

## 1 Introduction

Prevention of tumor metastasis is one of the principal targets for medical treatment of multiple cancers.^[^ ^1^, ^2^, ^3^ ^]^ The extremely sophisticated process and molecular mechanism led to the unsatisfactory progress in this area.^[^ ^4^, ^5^, ^6^, ^7^, ^8^ ^]^ Recently, the programmed necrosis (necroptosis) of vascular endothelial cells (ECs) induced by tumor cells (TCs) in blood system has been reported for the first time, which promotes TCs extravasation and metastasis.^[^ ^9^ ^]^ Consequently, blocking the necroptosis pathway could be a potential method to prevent tumor hematogenous metastasis.^[^ ^10^, ^11^ ^]^ Up to now, most studies in this field focus on the development of inhibitors of receptor‐interacting protein kinase 1 (RIPK1),^[^ ^12^, ^13^, ^14^, ^15^ ^]^ which is the key multifunctional protein involved in the regulation of necroptosis,^[^ ^16^ ^]^ but many known RIPK1 inhibitors are not suitable for clinical studies due to their suboptimal pharmacokinetic properties or side effects.^[^ ^17^ ^]^ Furthermore, Strilic et al. revealed for the first time that the binding of amyloid precursor protein (APP) to death receptor 6 (DR6) relieved the positive regulation of necroptosis pathway, and knockdown of either APP or DR6 results in a significant reduction of TC‐induced necroptosis as well as TCs transendothelial migration.^[^ ^9^ ^]^ This behavior suggests that the interaction between APP and DR6 provides a novel target for anti‐hematogenous metastatic therapies.

DR6 is broadly expressed by multiple cells including neuronal cells and ECs, and best known for its association with normal cell body death and axonal pruning.^[^ ^18^, ^19^, ^20^, ^21^ ^]^ Its extracellular domain consists of four cysteine‐rich domain (CRD) modules and followed by a transmembrane domain and cytoplasmic death domain.^[^ ^22^ ^]^ DR6 expressed on the surface of ECs could be activated by APP,^[^ ^23^, ^24^, ^25^ ^]^ another single‐pass transmembrane protein and abundantly expressed by TCs,^[^ ^26^, ^27^, ^28^, ^29^, ^30^, ^31^ ^]^ and subsequently initializes the necroptosis of ECs. The direct binding interface between DR6/APP was reported by Nikolov's group, which is formed by the first CRD module of DR6 and H1, H2 helices of APP‐E2 domain.^[^ ^24^ ^]^ This DR6/APP complex implies that targeting of the surface pocket in CRD1 of DR6 by exogenous chemicals may restraint the activation of necroptosis pathway to prevent tumor metastasis. To the best of our knowledge, there is currently no report about the inhibitor of DR6/APP interaction, and the highly potent inhibitor is still urgently needed.

**Figure 1:**
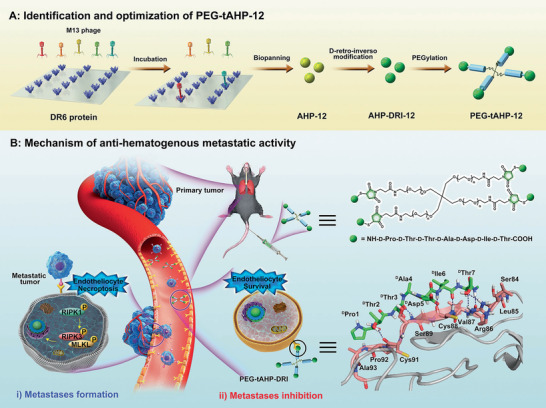
Schematic illustration of discovery of peptidomimetic–polymer inhibitor to DR6/APP interaction and its mechanism of inhibiting hematogenous metastasis. A) Identification and optimization of PEG‐tAHP‐12. Peptide AHP‐12 (yellow ball) is initially selected through 7‐amino acid peptide library expressed on M13 phage, which is further underwent structure optimization via d‐retro‐inverso modification to generate peptidomimetic AHP‐DRI‐12 (green ball); Finally, the polymer–peptidomimetic PEG‐tAHP‐12 is prepared through multiple conjugation of screened peptidomimetic agent with 4‐arm PEG_5k_. B) Mechanism of anti‐hematogenous metastatic activity. The TCs from the primary tumor could induce the ECs necroptosis after the binding of APP expressed on the TCs to DR6 on ECs in the circulatory system, which lead to TCs extravasation and metastasis. The polymer–peptidomimetic PEG‐tAHP‐12 with high binding potency to DR6 (explained by molecular docking assay) could effectively block the DR6/APP interaction which successfully inhibit the ECs necroptosis and achieve the metastases inhibition.

Herein, we provide what to our knowledge the first class of DR6/APP interaction inhibitor, generated via three rationally designed combined strategies. The structure‐based drug design utilizing a random heptapeptide phage display library was first adopted to obtain 24 peptides with good affinity to DR6, and then structure optimization of these peptides was conducted through d‐retro‐inverso modification that afforded eight peptidomimetic with stronger bind potency and better pharmacokinetic character. Furthermore, the conjugate PEG‐tAHP‐DRI was achieved as the most potent inhibitor by exploiting the multi‐arm polymer conjugation with screened peptidomimetic, which not only displays strongest affinity to target, but also has satisfactory serum half‐life. As we expected, this conjugate inhibitor shows excellent anti‐hematogenous metastatic effect both in vitro and in vivo owing to its high binding affinity to DR6 and long‐term effect in the circulatory system. Moreover, PEG‐tAHP‐DRI displays no apparent toxicity and no detrimental effects to the cardiac, liver, and renal, and it was natively inert to DR6‐related signaling pathway, which shed light on the safe clinical application of PEG‐tAHP‐DRI. Taken together, this study presented a rather promising DR6‐targeting polymer‐conjugate agent for future anti‐hematogenous metastatic therapies (**Figure** **1**).

## 2 Results and Discussion

### 2.1 PEG‐tAHP‐DRI Is Identified as the Strongest Inhibitor to DR6/APP Interaction

**Figure 2:**
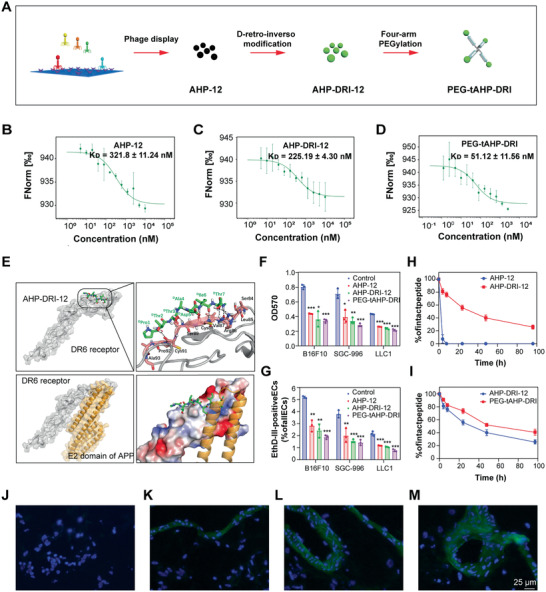
PEG‐tAHP‐DRI is identified as the strongest inhibitor to DR6/APP interaction. A) Schematic identification of PEG‐tAHP‐DRI through three combined strategies. B–D) The binding curve of AHP‐12, AHP‐DRI‐12, or PEG‐tAHP‐DRI to DR6 protein was measured by MST, *K*_D_ = 321.8 ± 11.24 × 10^−9^ m, *K*_D_ = 225.19 ± 4.30 × 10^−9^ m, and *K*_D_ = 51.12 ± 11.56 × 10^−9^ m, respectively. E) The binding mode of AHP‐DRI‐12 with DR6 receptor. Residues involved in binding are represented by green sticks (AHP‐DRI‐12) and pink sticks (DR6); hydrogen bonds are denoted by black dashed lines. F) Cell attachment of different TCs to culture plate coated with DR6 protein after a treatment with AHP‐12, AHP‐DRI‐12, or PEG‐tAHP‐DRI. G) HUVECs necroptosis upon exposure to different TCs after a treatment with AHP‐12, AHP‐DRI‐12, or PEG‐tAHP‐DRI. H,I) Degradation of AHP‐DRI‐12 and PEG‐tAHP‐DRI in 25% rat serum. J–M) Representative images of staining for DAPI (blue) of lung sections after intravenous injection of J) vehicle, K) FITC (green) labeled AHP‐12, L) AHP‐DRI‐12, or M) PEG‐tAHP‐DRI. Data are means ± SD (*n* = 3), and analyzed with GraphPad Prism 8.0. *p < 0.05, **p < 0.01, and ***p < 0.005 calculated by unpaired two‐tailed Student's *t*‐test. NS indicates *p* > 0.05.

Necroptosis is tightly regulated by endothelial DR6‐mediated signaling pathways, development of inhibitor to DR6–APP interaction could be a promising strategy for regulation of hematogenous metastatic dissemination of cancer that induced by the necroptosis of vascular ECs.^[^ ^9^ ^]^ To meet the requirements of strong binding potency and long serum half‐life for anti‐hematogenous metastatic agents, polymer–peptidomimetic conjugate was selected due to the comprehensive advantages of peptides and biocompatible polymers.^[^ ^32^, ^33^ ^]^ In this work, we fabricated an effective and long acting polymer–peptidomimetic agent for anti‐hematogenous metastatic therapies, which was prepared by multiple conjugation of screened peptidomimetic agent with 4‐arm PEG_5k_ (**Figure** **2**A). The synthesized conjugate PEG‐tAHP‐DRI not only displays excellent binding potency to DR6 due to rational structure‐based drug design strategy, but also demonstrates satisfactory proteolytic stability owing to the retro‐inverso modification and multiple conjugation with biocompatible polymer.

Initially, a random heptapeptide phage display library was adopted for screening the amino acid sequences that bind to the interface between DR6‐APP, and 24 different sequences were originally obtained. Subsequently, we synthesized these peptides by typical solid‐phase peptide synthesis (SPPS) approach and further screened their binding affinity to DR6 through microscale thermophoresis (MST). The results showed that AHP‐12 (TIDATTP), with the *K*_D_ value of 321.8 ± 11.24 × 10^−9^ m, was the best inhibitor of these synthetic heptapeptides (Figure 2B; Table S1 and Figure S1, Supporting Information). Next, to optimize the potency of these inhibitors, both d‐replace and retro‐inverso modification strategies were utilized. Remarkably, to verify the effectiveness of this modification, other than AHP‐12 with excellent potency, AHP‐03, AHP‐10, and AHP‐13 with medium potency were all subjected to this modification. All the eight peptidomimetic inhibitors were evaluated for their potency (Table S2, Supporting Information). To our delight, the d‐retro‐inverso isomer of AHP‐12, AHP‐DRI‐12, demonstrated the best optimized data in MST with a *K*_D_ value of 225.19 ± 4.3 × 10^−9^ m (Figure 2C). In addition, to further improve the potency and prolong the serum half‐life, conjugation of the small‐sized peptidomimetic with multifunctional 4‐arm PEG was investigated. Conceivably, polymer‐drug conjugates have shown prominent advantages compared with the corresponding parent drugs in clinical trials, including stronger therapeutic efficacy, less side effect, better pharmacokinetic property, and ease of drug administration.^[^ ^34^, ^35^, ^36^ ^]^ The PEGylated tetrameric AHP‐DRI‐12 (PEG‐tAHP‐DRI) was prepared by conjugating of one 4‐arm PEG_5k_ maleimide with four modified AHP‐DRI‐12 molecules at the C‐terminal of additional Cys residue (Figure S2, Supporting Information), and PEG‐tAHP‐DRI demonstrated almost fourfold greater binding affinity (*K*_D_ = 51.12 × 10^−9^ m) to DR6 than AHP‐DRI‐12 (Figure 2D), which could be rationalized by multivalent effect based on its tetra‐conjugated chemical structure.

Molecule docking study was performed to investigate the detailed interaction of the AHP‐DRI‐12 in DR6. Analyzing the binding mode of the AHP‐DRI‐12, it was found that the peptidomimetic could bind into the pocket where APP interacts with DR6 receptor and formed effective interaction with DR6 (Figure 2E). The AHP‐DRI‐12 occupied part of the binding area where APP interacts with DR6 receptor, which indicated the AHP‐DRI‐12 could block TCs‐induced necroptosis of ECs. Examining the interaction detail between AHP‐DRI‐12 and DR6, it showed that intensive hydrogen bonds were formed which effectively stabilized the interaction between ligand and DR6, including ^D^Thr7 with Arg86 in DR6, ^D^Ile6, ^D^Thr3, and ^D^Thr2 with backbone of DR6‐CRD1. Moreover, hydrophobic interactions between ^D^Thr7 and Leu85, ^D^Ile6 and Val87, and ^D^Thr3 and Ser89 also significantly contributed to stabilization of this binding mode. As description of the docking result, the crucial DR6/APP binding sites,^[^ ^24^ ^]^ such as Arg86, Leu85, and Val87, were tightly occupied by exogenous peptidomimetic that rationalized the molecular mechanism of anti‐hematogenous metastasis activity of the AHP‐DRI‐12 and PEG‐tAHP‐12.

The cell attachment assay was conducted to validate the DR6/APP interaction blocking effect by treating different TCs (B16F10, SGC‐996, and LLC1) with these DR6‐targeting peptide/peptidomimetic. The most obvious disruption of attachment between TCs and DR6‐coated plates was observed after treatment with PEG‐tAHP‐DRI, which indicated the conjugate possesses more positive effect on the blockade of DR6/APP interaction than AHP‐12 and AHP‐DRI‐12 (Figure 2F; Figures S3A and S4A, Supporting Information). To further investigate the effect of these ligands on inhibiting TCs‐induced HUVECs necroptosis, co‐culture assays of human umbilical vein ECs (HUVECs) and TCs with these ligands were conducted as a complementary approach. Three TC lines, which could stably express the green fluorescent protein (GFP) (Figure S5A, Supporting Information), were co‐cultured with HUVECs, and dual staining with Hoechst and ethidium‐homodimer‐III (EthD‐III) visually showed the ratio of TCs‐induced necroptotic HUVECs (Figures S3B, S4B, and S5B, Supporting Information). As we expected, PEG‐tAHP‐DRI demonstrated the strongest potent inhibitory effect on HUVECs necroptosis (Figure 2G).

The proteolytic stability of peptide/peptidomimetic and the conjugate was evaluated through investigating the pharmacokinetic property via incubating with 25% fresh rat serum. It was found that original AHP‐12 demonstrated fast degradation and most intact peptide disappeared within 4 h. In contrast, AHP‐DRI‐12 only displayed slight degradation under the same condition, which suggested that the d‐retro‐inverso modification could significantly alter the pharmacokinetic property of designed peptide agent (Figure 2H). Moreover, PEG‐tAHP‐DRI exhibited obviously enhanced proteolytic resistance than AHP‐DRI‐12, especially after 8 h incubation period, which could be attributed to the steric hindrance of branched PEGylation (Figure 2I). In addition, DAPI staining of lung frozen sections was further utilized to investigate the binding potency and pharmacokinetic properties of these ligands in vivo. After injection of fluorescein isothiocyanate (FITC)‐labeled ligand, strong fluorescence intensity and uniform distribution were observed on surface of blood vessels (Figure 2J–M), which visually proved that the polymer–peptidomimetic conjugate PEG‐tAHP‐DRI not only possesses superior strong binding potency, but also the best proteolytic stability.

### 2.2 PEG‐tAHP‐DRI Displayed Significant Anti‐Hematogenous Metastatic Effect In Vitro

**Figure 3:**
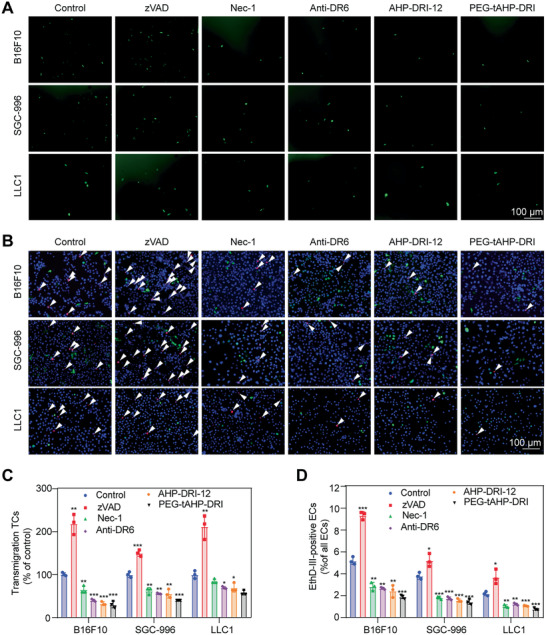
PEG‐tAHP‐DRI inhibited TC‐induced HUVECs necroptosis in vitro. A) TCs transmigration over the HUVEC monolayer after a treatment with zVAD, Nec‐1, anti‐DR6 antibody, AHP‐DRI‐12, or PEG‐tAHP‐DRI. B) HUVECs necroptosis after a treatment with zVAD, Nec‐1, anti‐DR6 antibody, AHP‐DRI‐12, or PEG‐tAHP‐DRI. C) Quantification of transmigration TCs after a treatment with zVAD, Nec‐1, anti‐DR6 antibody, AHP‐DRI‐12, or PEG‐tAHP‐DRI. D) Quantification of necroptosis HUVECs after a treatment with zVAD, Nec‐1, anti‐DR6 antibody, AHP‐DRI‐12, or PEG‐tAHP‐DRI. Data are means ± SD (*n* = 3), and analyzed with GraphPad Prism 8.0. *p < 0.05, **p < 0.01, and ***p < 0.005 calculated by unpaired two‐tailed Student's *t*‐test. NS indicates *p* > 0.05.

To determine whether these ligands inhibit TCs transendothelial migration, we utilized assay of transendothelial migration in vitro to assess the regulation ability of theses peptidomimetic agents, which measures the number of TCs that migrated through the tight HUVECs monolayer (**Figure** **3**A,C). This in vitro assay has been proven to be a reproducible and reliable model to analyze the behavior of ECs and TCs in vivo. It was found that both AHP‐DRI‐12 and PEG‐tAHP‐DRI at 1 × 10^−6^ m significantly attenuated TCs transendothelial migration compared with the control. In contrast, increased number of migrated TCs was observed in z‐VAD‐fmk (zVAD)‐treated control, which could be rationalized that zVAD could act as a transducer of necroptotic signaling to shift apoptosis to necroptosis.^[^ ^37^ ^]^ It was worth noting that though the RIPK1 inhibitor (necrostatin‐1 (Nec‐1))^[^ ^12^ ^]^ and anti‐DR6 antibody demonstrated similar potency, but the strongest inhibitory effect was still exhibited by peptidomimetic conjugate PEG‐tAHP‐DRI. Co‐culture assay was also conducted to evaluate the inhibitory effects of the designed ligands, the PEG‐tAHP‐DRI group showed much less amount of necroptotic HUVECs than all AHP‐DRI‐12 group, Nec‐1‐treated, and anti‐DR6‐treated controls in different TC lines (Figure 3B,D). Collectively, the data in both transendothelial migration and co‐culture assay indicated that PEG‐tAHP‐DRI could effectively inhibit TC transendothelial migration in vitro, which was even better than RIPK1 inhibitor and anti‐DR6 antibody.

### 2.3 PEG‐tAHP‐DRI Exhibits Excellent Anti‐Hematogenous Metastatic Effect In Vivo

**Figure 4:**
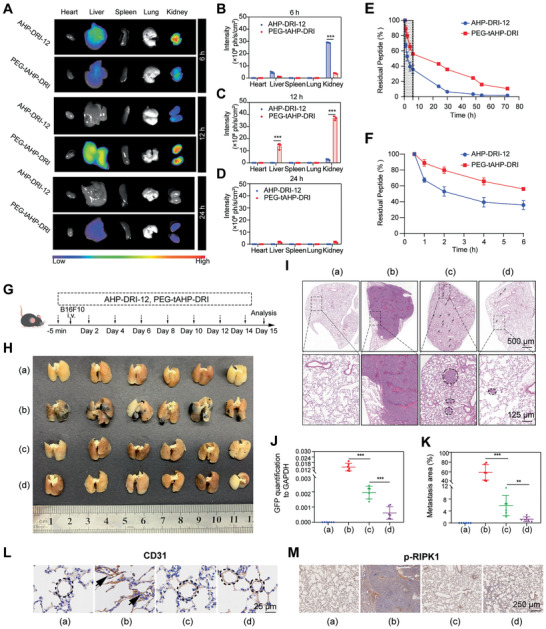
PEG‐tAHP‐DRI exhibited efficient anti‐metastatic ability and favorable pharmacokinetic properties. A) Fluorescence images of organs after the indicated intravenous injection for 6 or 12 h. B–D) Quantitative analysis of the mean fluorescence intensity of FITC in each group. E) Pharmacokinetics of AHP‐DRI‐12 and PEG‐tAHP‐DRI after the intravenous injection. F) Magnification of percentage of residual peptide from 0 to 6 h. Data are means ± SD (*n* = 3). Data are analyzed with GraphPad Prism 8.0. *p < 0.05, **p < 0.01, and ***p < 0.005 calculated by unpaired two‐tailed Student's *t*‐test. NS indicates *p* > 0.05. G) Experimental design. H) Photographs of the lungs 15 day after intravenous injection of B16F10. a) Saline, b) B16F10+saline (vehicle), c) B16F10+AHP‐DRI‐12, and d) B16F10+PEG‐tAHP‐DRI. I) H&E staining of lung sections. J) Quantification of GFP expression level of lungs. K) Metastasis areas of lungs were quantified. L,M) Immunohistochemical images of the vascular integrity analysis on B16F10 tumor‐bearing mice treated with different preparations after 15 days. The brown cells shown in the circles were stained by CD31. p‐RIPK1 staining of lung sections to evaluate metastasis. Data are means ± SD (*n* = 6).

As mentioned above, the ideal peptidomimetic inhibitor for anti‐hematogenous metastatic therapies should not only possess strong affinity to target, but also have long serum half‐life in the circulatory system. Consequently, both pharmacokinetic properties and anti‐hematogenous metastasis effect of the polymer‐peptidomimetic conjugate in vivo were further investigated. In vivo fluorescence imaging showed that within 6 h after injection, both AHP‐DRI‐12 and PEG‐tAHP‐DRI were detected to accumulate mostly in kidney and slightly in liver, and the polymer conjugate PEG‐tAHP‐DRI exhibited much lower distribution intensity. After 12 h, PEG‐tAHP‐DRI showed much higher distribution in kidney and liver than AHP‐DRI‐12 and the small peptidomimetic AHP‐DRI‐12 could not be detected in liver. Furthermore, there was almost no fluorescence distribution in these tissues after 24 h (**Figure** **4**A–D). Subsequently, the peptidomimetic levels in rat serum were measured the half‐life (T_1/2_) of AHP‐DRI‐12 and PEG‐tAHP‐DRI were 7.8 and 11.1 h, respectively, and the area under the curve (AUC_0‐72_) of PEG‐tAHP‐DRI was significantly larger than that of AHP‐DRI‐12 (Figure 4E,F). The above results indicated that both d‐retro‐inverso modification and multiple conjugation could contribute to the low clearance rate of the synthetic agent in the blood circulation. Collectively, PEG‐tAHP‐DRI possesses longer serum half‐life than AHP‐DRI‐12 due to the increase in the molecular weight by branched PEG, and it was metabolized by the both liver and kidney.

Subsequently, the B16F10, 4T1, and CT26 lung metastasis models as well as the spontaneous lung metastasis of 4T1 orthotopic model were utilized to study the in vivo anti‐hematogenous metastasis effect. After the pretreatment of peptidomimetic agents, the mice were intravenously injected with TCs (B16F10, 4T1, and CT26) to build lung metastasis models (Figure 4G; Figures S6A and S7A, Supporting Information). Mice were sacrificed and their lungs were obtained for observation and histological analysis. To our delight, the PEG‐tAHP‐DRI‐treated group showed the best result with least amount of pulmonary metastasis nodules, indicating an inhibitory effect of PEG‐tAHP‐DRI on metastasis of TCs to the lungs in mice (Figure 4H; Figures S6B and S7B, Supporting Information). For the evaluation of this efficacy in spontaneous lung metastasis of orthotopic tumor model, 4T1 cells were utilized to develop orthotopic model (Figure S8, Supporting Information). As we expected, PEG‐tAHP‐DRI could efficiently restrain spontaneous metastasis to lungs, while there was no significant difference in orthotopic tumor size of the different groups. Moreover, lungs were sliced and examined visually by hematoxylin and eosin (H&E) staining, and the PEG‐tAHP‐DRI‐treated mice showed less and smaller metastatic loci and normal morphology like the saline‐treated mice (Figure 4I,K; Figures S6C,D and S7C,D, Supporting Information). Consistently, PEG‐tAHP‐DRI‐treated mice demonstrated less expression level of GFP mRNA in lungs than both vehicle‐treated and AHP‐DRI‐12‐treated mice (Figure 4J), which could be attributed to the prolonged plasma half‐life of the polymer–peptidomimetic agent.

In addition, the influences of PEG‐tAHP‐DRI on the vasculature ECs were investigated by utilizing CD31 staining and *p*‐RIPK1 staining (Figure 4L,M). After staining with endothelial marker CD31, the vascular endothelium with an apparent disorganized morphology was observed in lungs of vehicle‐treated mice. In contrast, the pulmonary vessels of the other three groups, saline‐treated mice, AHP‐DRI‐12‐treated, and PEG‐tAHP‐DRI‐treated mice, still remained relatively intact and orderly. Meanwhile, *p*‐RIPK1 immunohistochemical analysis exhibited a dramatic decrease of necroptosis in lungs of both AHP‐DRI‐12‐ and PEG‐tAHP‐DRI‐treated mice, and the conjugate exhibited superior anti‐hematogenous metastatic effect. Taken together, these results demonstrated that PEG‐tAHP‐DRI could significantly suppress hematogenous metastasis in vivo, thus highlighting PEG‐tAHP‐DRI as a DR6/APP inhibitor with promising potential for the development of anti‐hematogenous metastatic therapeutics. Taken together, all these results indicated that PEG‐tAHP‐DRI was a promising long‐acting polymer–peptidomimetic anti‐hematogenous metastatic agent.

### 2.4 PEG‐tAHP‐DRI Shows No Obvious Toxicity and Native Activity to Apoptosis/Necroptosis Pathway In Vitro

As shown in the above study, extensive evidence has verified that PEG‐tAHP‐DRI could restrain the activation of necroptosis pathway by targeting the protein–protein interaction (PPI) interface of APP and DR6. Thus, the detailed physiological effects of this conjugate are quite important to its further clinic application and a series of preliminary safety evaluations was conducted. Specifically, DR6 is a member of tumor‐necrosis factor receptor superfamily and plays a critical role in regulation of cell number such as elimination of virus‐infected or harmful cells.^[^ ^18^, ^19^, ^20^, ^38^, ^39^ ^]^ Theoretically, either apoptosis or necroptosis could be induced by the stimulation of DR6 via two distinct pathways,^[^ ^40^, ^41^, ^42^ ^]^ thus the safety assessment of PEG‐tAHP‐DRI is vital for the development of anti‐hematogenous metastasis agent.

**Figure 5:**
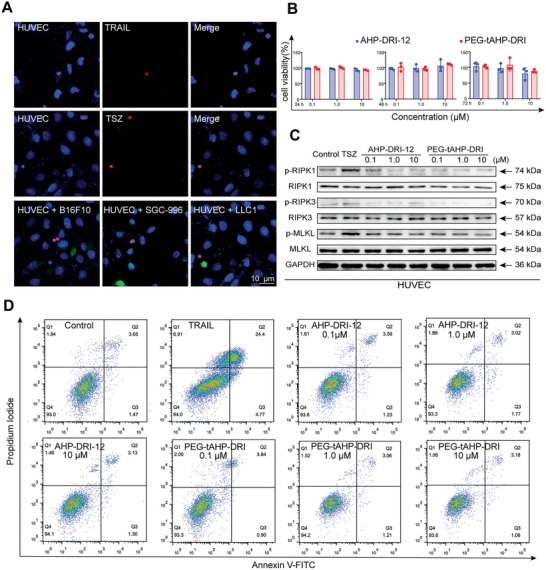
PEG‐tAHP‐DRI had no effect on HUVECs function in vitro. A) Confocal image of HUVECs in the presence of TRAIL, TSZ, or TCs (GFP‐B16F10, GFP‐SGC‐996, and GFP‐LLC1). B) HUVECs viability after incubated with different concentration peptides at 24, 48, and 72 h. C) Expression level of RIPK1, RIPK3, and MLKL and their phosphorylated states were measured after a treatment with TSZ, AHP‐DRI‐12, or PEG‐tAHP‐DRI. D) Apoptosis analysis of HUVECs after a treatment with TRAIL, AHP‐DRI‐12, or PEG‐tAHP‐DRI (0.1 × 10^−6^, 1.0 × 10^−6^, and 10 × 10^−6^ m). Data are means ± SD (*n* = 3) and analyzed with GraphPad Prism 8.0.

The cytotoxicity of PEG‐tAHP‐DRI was assessed on HUVECs, and cell viability had remained essentially unchanged even at the high concentration (10 × 10^−6^ m) (**Figure** **5**B). Furthermore, examination of the effect of PEG‐tAHP‐DRI in apoptosis induction was conducted by utilizing TNF‐related apoptosis‐inducing ligand (TRAIL) as positive control. As expected, the results showed that 30% of TRAIL‐treated HUVECs underwent apoptosis and several typical apoptotic features in morphology were observed, while other groups (including PBS control) demonstrated similarly low rates of apoptosis (Figure 5A,D). To determine whether PEG‐tAHP‐DRI could natively activate necroptosis pathway, such as stimulating of DR6 or inducing phosphorylation of its downstream signaling protein RIPK1, RIPK3, or MLKL, western blot experiments were carried out (Figure 5C), and the results demonstrated that both synthetic peptidomimetic agents could not affect the expression levels of p‐RIPK1, p‐RIPK3, and p‐MLKL, while TNF‐*α*/Smac‐mimetic/z‐VAD‐fmk (TSZ) treatment could activate the necroptosis pathway with typical necroptotic feature (Figure 5A). Therefore, PEG‐tAHP‐DRI is an inactive ligand and could not initialize necroptosis pathway in vitro.

### 2.5 PEG‐tAHP‐DRI Is Natively Inert to DR6‐Related Signaling Pathway In Vivo

**Figure 6:**
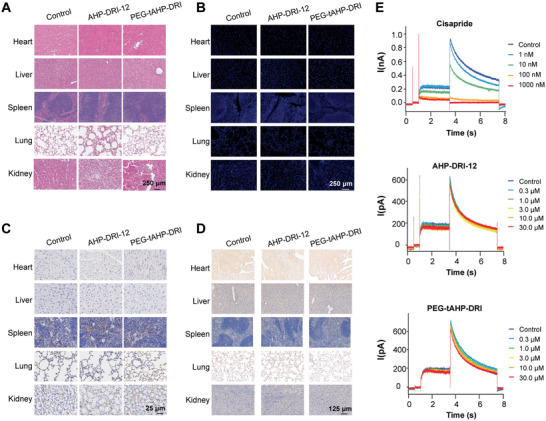
PEG‐tAHP‐DRI had no influence on DR6‐related signaling pathway and no cardiotoxicity. A) H&E staining of tissue sections (heart, liver, spleen, lung, and kidney) of healthy mice 15 days after being injected with AHP‐DRI‐12 or PEG‐tAHP‐DRI. B) Tissue sections (heart, liver, spleen, lung, and kidney) of healthy mice 15 days after being injected with AHP‐DRI‐12 or PEG‐tAHP‐DRI were stained with TUNEL. C,D) Tissue sections (heart, liver, spleen, lung, and kidney) of healthy mice 15 days after being injected with AHP‐DRI‐12 or PEG‐tAHP‐DRI were stained with C) CD31 and D) p‐RIPK1. E) Current change of hERG induced by cisapride, AHP‐DRI‐12, or PEG‐tAHP‐DRI.

To further corroborate the inherent inertness of PEG‐tAHP‐DRI to DR6‐related signaling pathway, healthy C57BL/6 mice intravenously injected with peptidomimetic agents were used as models for safety assessment of the anti‐hematogenous metastatic agents in vivo. Histological analysis of various tissues from two groups of ligand‐treated mice that were sacrificed on day 2 or 15, respectively, was performed (**Figure** **6**A; Figure S9A, Supporting Information). Tissues including heart, liver, spleen, lung, and kidney from peptidomimetic‐treated mice showed normal morphology like the saline‐treated mice by H&E staining. Collectively, no obvious increase in apoptotic cells was observed in PEG‐tAHP‐DRI‐treated mice by terminal dUTP nick end labeling (TUNEL) staining (Figure 6B; Figure S9B, Supporting Information). In addition, a series of immunohistochemistry analysis was carried out such as CD31 staining, and its results demonstrated that the PEG‐tAHP‐DRI could not cause damage to the vessels (Figure 6C; Figure S9C, Supporting Information). Moreover, the results of p‐RIPK1, p‐I*κ*B‐*α* (whether NF‐*κ*B is activated),^[^ ^43^ ^]^ and caspase 8 staining proved that downstream signaling pathway of DR6 could not be impacted by the PEG‐tAHP‐DRI (Figure 6D; Figure S9D–F, Supporting Information). These findings clearly verified that PEG‐tAHP‐DRI is natively inert to DR6‐related signaling pathway during its inhibition procedure of TC‐induced ECs necroptosis in vivo.

In addition, some common adverse effects suffered by frequently used medications were also evaluated. The whole‐cell patch clamp recording technique was used to record the whole‐cell hERG‐potassium to measure the cardiac toxicity of peptidomimetic agents on hERG potassium channel by utilizing cisapride as positive control (Figure 6E; Figure S10, Supporting Information). The results showed that both AHP‐DRI‐12 and PEG‐tAHP‐DRI had no significant inhibitory effect on hERG channels and would not increase the risk of arrhythmias. Meanwhile, the blood biochemical indexes of ligand‐treated mice sacrificed on day 2 or day 15 were tested to examine the influences of peptidomimetic agents to liver and kidney (Figure S11, Supporting Information). The results consistently proved that AHP‐DRI‐12 and PEG‐tAHP‐DRI could not cause any damage to liver or kidney. Taken together, all these results indicated that PEG‐tAHP‐DRI could effectively and selectively inhibit the DR6/APP interaction.

## 3 Conclusion

TC‐induced ECs necroptosis has been proved to be the important pathogenesis of tumor hematogenous metastasis, thus the inhibition of the ECs necroptosis in the context of tumor progression represents a promising therapeutic target.^[^ ^9^, ^44^, ^45^, ^46^, ^47^, ^48^, ^49^ ^]^ Recent findings about lung metastases of cancer cells in blood system provide a pioneering evidence that APP‐DR6 signaling pathway plays a critical role for ECs necroptosis and TCs extravasation, which indicates that inhibitors of DR6 are viewed as novel anti‐metastasis agents. However, identifying inhibitors of DR6/APP interaction with high binding affinity and desired pharmacokinetic property in blood system is extremely challenging. In current study, we discovered the ligand PEG‐tAHP‐DRI, with a multi‐arm PEGylated peptidomimetic conjugate chemical structure, is a novel necroptosis inhibitor that can effectively block the DR6/APP interaction both in vitro and in vivo and keep long‐term effects in blood system. This study not only demonstrates the feasibility of exploiting the DR6/APP interaction as anti‐metastatic therapeutic target for drug discovery, but also established an effective strategy in PPI inhibitor research for polymer–peptidomimetic conjugates and structure‐based drug design.

We used a 7‐amino acid peptide library expressed on M13 phage to rationally design a series of peptide inhibitors of DR6/APP PPI. These peptides and their d‐replace/DRI isomers were synthesized and their binding affinity to DR6 was evaluated via MST, which resulted in peptidomimetic AHP‐DRI‐12 as the optimal ligand. The binding model of AHP‐DRI‐12 to DR6 target was further analyzed by molecular modeling study, and the results revealed that function groups on both backbone and branched of the peptidomimetic could form intensive hydrogen bond and hydrophobic interactions, which effectively stabilized this binding model. It is noteworthy that innovative application of multi‐arm PEGylated strategy to the optimization of peptidomimetic inhibitors afforded conjugate PEG‐tAHP‐DRI as the most efficient inhibitor of DR6/APP interaction, which demonstrated *K*_D_ value of about 50 × 10^−9^ m and significantly prolonged serum half‐life. Consequently, the combined strategies of selection based on phage display library, d‐retro‐inverso modification, and multiple conjugation of small‐sized peptidomimetic with 4‐arm PEG is an effective approach to develop high‐potent and long‐acting peptide/peptidomimetic drugs.

Further in‐depth studies of PEG‐tAHP‐DRI were carried out including in vitro and in vivo bioactivities evaluation, preliminary pharmacokinetic characterization, and safety assessment. The polymer–peptidomimetic conjugate could efficiently inhibit DR6/APP interaction and attenuate the necroptotic cell death of ECs induced by TCs both in vitro and in vivo, resulting in significant suppression of hematogenous metastasis. Importantly, this ligand displayed a favorable pharmacokinetic profile with desired long‐time serum half‐life and no apparent toxicity in mice, and its native inertness to DR6‐related pathway was further confirmed both in vitro and in vivo. In conclusion, PEG‐tAHP‐DRI has a great potential for the development of novel anti‐hematogenous metastasis agent and deserves further research about clinic application.

## 4 Experimental Section

##### Materials, Cells, and Animals

Ph.D. Phage Display Library was purchased from New England Biolabs, Inc. (Beijing, China). Peptides were synthesized via SPPS using active ester chemistry to fluorenylmethyloxycarbonyl (Fmoc)‐protected amino acid to the deprotected resin. EthD‐III and Hoechst 33342 were purchased from Biotium, Inc. (Fremont, Canada). Monolith His‐Tag Labeling Kit RED‐tris‐NTA second generation was purchased from NanoTemper Technologies Co., Ltd. (Beijing, China). DR6 protein and anti‐DR6 were purchased from Sino Biology, Inc. (Shanghai, China). Nec‐1 and z‐VAD were purchased from Enzo Biochem (New York, USA). Recombinant human TNF‐*α* and recombinant human sTRAIL/Apo2L were purchased from Peprotech, Inc. (Rocky Hill, USA). Smac‐mimetic was purchased from Selleck (Shanghai, China). Antibodies for RIPK1, MLKL, p‐RIPK3, and p‐MLKL were purchased from Abcam (Cambridge, UK). Antibodies for RIPK3 and p‐RIPK1 were purchased from Peprotech, Inc. HRP‐labeled secondary antibodies were purchased from Cell Signaling Technology (MA, USA).

Cell lines B16F10, SGC‐996, LLC1, 4T1, CT26, and HUVECs were provided by State Key Laboratory of Oncogenes and Related Genes. The cells were cultured by following the instructions. C57BL/6, Balb/c mice, and SD rat were purchased from Shanghai SLAC Laboratory Animal Co., Ltd. (Shanghai, China) and kept under SPF conditions. All animal experiments were carried out in accordance with guidelines evaluated and approved by the ethics committee of Shanghai Jiao Tong University.

##### Phage Display Assay

The bio‐panning was performed by a phage display library (about 10^9^), as described previously.^[^ ^50^ ^]^ Briefly, DR6 protein was dissolved in 0.1 m NaHCO_3_ buffer and then 150 µL DR6 protein solution was added into each well of a 96‐well microtiter plate. The plate was kept at 4 ℃ overnight to immobilize DR6 protein onto 96‐well plate. Next day, the DR6 protein solution was gently removed from the 96‐well plate. Then, the wells were washed with TBS (50 × 10^–3^ m Tris‐HCl (pH 7.5) and 150 × 10^–3^ m NaCl), and a blocking solution, 1% albumin from bovine serum albumin (BSA) in TBS, was added to the wells and incubated for 2 h with agitation at 4 ℃ to make the surface neutralized and block nonspecific adsorption sites. After removal of the blocking buffer, the wells were washed three times with 200 µL TBS to prepare for the following biopanning process. Then, 10 µL 7‐amino acid‐long random peptide library was added in 90 µL TBST and then the solution was added in 96‐well plate that was coated with DR6 protein. Following incubation at 25 ℃ for 4 h, the wells were washed ten times to remove the unbound phages with TBST. Acid elution (50 µL of 50 × 10^–3^ m glycine–HCl, pH 2.0) was used to elute the bound phages. Then phosphate buffer (pH 7.5) was applied for neutralization of the solutions. In the first round, each well was washed ten times with no incubation. In the following rounds, the wells were washed 12 times with incubation for amounts of time. After three rounds of biopanning, individual clones were amplified and sequenced.

##### General Procedure for Solid‐Phase Synthesis of Peptides and Peptidomimetics

All peptides were synthesized via SPPS using active ester chemistry to Fmoc‐protected amino acid to the deprotected resin. Briefly, each peptide was coupled using 2‐(7‐aza‐1*H*‐benzotriazole‐1‐yl)‐1,1,3,3‐tetramethyluronium hexafluorophosphate as a coupling reagent and *N*‐ethyldiisopropylamine as a base in *N*,*N*‐dimethylformamide (DMF) for 1 h. Removal of Fmoc‐protecting group after each coupling step was carried out by 20% piperidine in DMF. Peptides were cleaved from the resin with mixture of the HFIP and DCM (20:80, vol/vol) for 1 h. Following filtration, the resulting cleavage solutions were concentrated in vacuo and purified by reversed‐phase high‐performance liquid chromatography to give the purified product peptides, and their integrity was confirmed by negative ion electrospray ionization mass spectrometry. Analytical data are provided in Figures S12 and S13, Supporting Information, and all peptides/peptidomimetics that were tested were >95% pure.

##### General Procedure for Synthesis of PEG‐tAHP‐DRI

AHP‐DRI‐12 with additional Cys residue at the C‐terminal and 4‐arm PEG_5k_ maleimide was dissolved in PBS (0.1 m, pH = 8) at a molar ratio of 8:1. The reaction mixture was stirred for 4 h at room temperature and then dialyzed in a Slide‐A‐Lyzer dialysis cassette (Thermo Fisher Scientific, Shanghai, China) (MWCO: 3500 Da) against water. The dialysate containing the pure product was lyophilized. The products were confirmed by nuclear magnetic resonance (^1^H NMR) spectra using a Bruker Avance 500 (500 MHz) spectrometer (Beijing, China). DLS analysis was used to measure the hydration dynamic radius of PEG‐tAHP‐DRI in solution and the results showed that size distribution of PEG‐tAHP‐DRI molecules is ≈10 nm (Figure S2D, Supporting Information). HPLC analysis showed the polymer–peptidomimetic conjugate has a high purity (Figure S2E, Supporting Information).

##### *K*_D_ Values of DR6‐Targeting Peptides for the Binding to DR6 Protein Were Measured by MST

For labeling DR6 protein with Red‐tris‐NTA second‐generation dye, equal volume of DR6 protein (200 × 10^–9^ m) and dye (100 × 10^–9^ m) were mixed. The solution was incubated for 30 min at room temperature and centrifuged for 10 min at 4 ℃ and 15 000 × *g*. The DR6 protein was labeled and ready for the binding assay. Prepare 25 µL of the DR6‐targeting peptides at 20 × 10^–6^ m and add 10 µL of the PBS‐T into the PCR tubes 2–12. Transfer 20 µL DR6‐targeting peptides into the PCR tube 1 and then transfer 10 µL of the DR6‐targeting peptides from tube 1 to tube 2 with a pipette and mix by pipetting up and down multiple times. Transfer 10 µL to tube 3 and mix. Repeat the procedure for tube 4–12. Add 10 µL of labeled protein to each tube (1–12) and mix by pipetting. The final DR6 protein concentration was 50× 10^−9^ m. Load the capillaries and measure the samples by NanoTemper Monolith NT.015.^[^ ^51^, ^52^ ^]^

##### Cell Attachment Assay

DR6 protein in 50 µL PBS (50 × 10^–9^ m) was added into 96‐well plates. The 96‐well plates were incubated for 2 h at 37 °C, followed by drying overnight at 4 °C. Next day, the 96‐well plates were warmly washed three times with PBS, and then blocked by PBS containing 1% BSA for 1 h at 37 °C. The plates were washed three times with PBS. TCs were suspended in serum‐free DMEM at a density of 3 × 10^5^ cells mL^–1^; 50 µL of the cell suspension was added to wells coated with the DR6‐targeting peptides. Different concentrations of DR6‐targeting peptides or PBS were added into the plates. After incubation at 37 °C for 1 h, non‐adherent cells were gently washed away with PBS, fixed with 4% paraformaldehyde at room temperature for 30 min, washed, stained with 5% crystal violet 50 µL per well for 10 min, gently washed with PBS, and decolorized with 33% acetic acid. The attachment cell number was measured by a microplate reader.^[^ ^53^ ^]^

##### Molecular Docking Study

The PDB file about crystal structure of DR6 (PDB ID:4YN0) was downloaded from Protein Bank on www.pdb.org. 3D structure model of the peptidomimetic AHP‐DRI‐12 was built by schrodinger2015 and the AHP‐DRI‐12 was docked into DR6 using glide module^[^ ^54^, ^55^ ^]^ in schrodinger2015.

##### Construction of GFP Stably Expressed TCs

The prepackaged lentivirus was purchased from Genomeditech Co., Ltd. (Shanghai, China). Briefly, TCs (1 × 10^5^) including B16F10, SGC‐996, and LLC1 were cultured overnight. Then, the culture medium was replaced with medium containing 5 µg mL^–5^ polybrene and lentivirus. After 24 h, the transduction mixture was replaced with fresh culture medium and the TCs were incubated for additional 24 h. Finally, infection efficiency was tested by fluorescence microscope.

##### Co‐Culture of TCs (B16F10, SGC‐996, and LLC1) and HUVECs

For co‐culture experiment, HUVECs (1 × 10^5^) were cultured for 24 h in six‐well plates and 5 × 10^4^ GFP‐expressing TCs (GFP‐B16F10, GFP‐SGC‐996, and GFP‐LLC1) were added alone or in the presence of the indicated substances onto the HUVECs monolayer and cultured for 24 h: peptides (1 × 10^–6^ m), Nec‐1 (30 × 10^–6^ m), and zVAD (100 × 10^–6^ m). Then, cells were stained with Hoechst 33342 and EthD‐III, and observed by fluorescence microscope. Cell number was determined by counting Hoechst‐33342‐positive cells, and cell death was determined by counting EthD‐III‐positive nuclei.^[^ ^9^ ^]^

##### Transendothelial Migration

HUVECs (1 × 10^5^) in DMEM were added into 24‐well plate transwell upper chambers and the medium was changed every 2 days. Using the EVOM resistance tester, the transendothelial resistance was measured daily. When the TEER resistance value was >200 Ωcm^2^, the cells grew into a monolayer of tight connection. 2 × 10^5^ GFP‐TCs were added into upper chambers. One milliliter DMEM containing 10% FBS was added into lower chambers overnight. AHP‐DRI‐12 and PEG‐tAHP‐DRI (1 ×10^–6^ m AHP‐DRI‐12), Nec‐1 (30 × 10^–6^ m), and zVAD (100 × 10^–6^ m) were added in upper chambers, respectively. After incubation for 48 h, cells in lower chambers were observed by fluorescence microscope.^[^ ^56^ ^]^

##### DAPI Staining of Lung Sections

FITC‐labeled AHP‐DRI‐12 and PEG‐tAHP‐DRI were injected intravenously and 30 min later, lungs were isolated to prepare frozen sections. The frozen sections were stained with DAPI. The representative images were taken on a confocal microscope.

##### In Vivo Anti‐Metastasis Assay

2 × 10^5^ GFP‐B16F10 cells, 2 × 10^5^ 4T1, or 2 × 10^5^ CT26 in 100 µL PBS were injected to the lateral tail vein of mice, respectively. The mice were intravenously injected with saline, AHP‐DRI‐12, and PEG‐tAHP‐DRI (0.2 µg g^–1^ AHP‐DRI‐12) shortly before and 24 h after injection of B16F10. Each formulation was administered every 2 days for a total of seven times. After 15 days, mice were sacrificed and perfused with PBS and 4% paraformaldehyde. The hearts, livers, spleens, lungs, and kidneys were collected for H&E staining and immunohistochemistry analysis. Lung metastases were analyzed by quantitation of GFP and metastases nodes.

The spontaneous lung metastasis of orthotopic breast tumor model was established via injection of 4T1 cells into the fourth inguinal mammary gland of Balb/c female mice. The mice were intravenously injected with saline, AHP‐DRI‐12, and PEG‐tAHP‐DRI (0.2 µg g^–1^ AHP‐DRI‐12), respectively, every 2 days for total of 14 times. Mice were sacrificed on day 30 and their lungs were collected for observation and histological analysis.

##### In Vitro Safety Analysis of DR6‐Targeting Peptides

To evaluate the cytotoxicity of DR6‐targeting peptides, MTT assay was conducted. Briefly, 4 × 10^3^ HUVECs were added in 96‐well plates and grown overnight before incubating with AHP‐DRI‐12 and PEG‐tAHP‐DRI (0.1 × 10^–6^, 1.0 × 10^–6^, 10 ×10^–6^ m AHP‐DRI‐12). After incubating for 24, 48, and 72 h, 100 µL of 0.5 mg mL^–1^ MTT was added to each well, and the plates were incubated at 37 °C for 4 h. After the removal of the supernatant, the precipitate was dissolved in 150 µL dimethyl sulfoxide, and the absorbance was measured at 490 nm using a microplate reader.

To evaluate the number of apoptotic cells after treatment of AHP‐DRI‐12 and PEG‐tAHP‐DRI, 5 × 10^5^ HUVECs were seeded in six‐well plates and treated with rhTRAIL (100 ng mL^–1^, Peprotech) or AHP‐DRI‐12 and PEG‐tAHP‐DRI (0.1 × 10^–6^, 1.0 × 10^–6^, 10 × 10^–6^ m AHP‐DRI‐12). After incubating for 24 h, HUVECs were harvested and resuspended in 100 µL binding buffer, and then stained with FITC‐labeled annexin V and propidium iodide (PI) (BD Biosciences) according to the manufacturer's instructions. The apoptotic number of HUVECs was measured by flow cytometry.

Western blot assay was conducted to examine the expression level of RIPK1, RIPK3, MLKL, and their phosphorylated states that were downstream protein of DR6. Briefly, 5 × 10^5^ HUVECs were added in six‐well plates and grown overnight. Next day, HUVECs were treated with TSZ, AHP‐DRI‐12, and PEG‐tAHP‐DRI (0.1 × 10^–6^, 1.0 × 10^–6^, 10 × 10^–6^ m AHP‐DRI‐12), respectively. After incubating for 24 h, HUVECs were washed with PBS, harvested, and mixed with 150 µL cell lysate buffer/well (Beyotime, China). Protein samples were collected after incubation at 4 ℃ for 30 min. Protein samples were centrifuged at 4 °C and 12 000 rpm for 10 min, and the supernatant was collected as samples. Each sample was then separated by 12% SDS–PAGE, followed by transferring to polyvinylidene difluoride membranes. Finally, samples were incubated with antibodies. HRP‐labeled secondary antibodies were added and measured using an imaging system.

##### In Vivo Safety Analysis of AHP‐DRI‐12 and PEG‐tAHP‐DRI

For evaluating the toxicity of peptides in vivo, 6 week old healthy mice were intravenously injected with AHP‐DRI‐12 and PEG‐tAHP‐DRI (0.2 µg g^–1^ AHP‐DRI‐12). After 2 and 15 days, mice were sacrificed and serum samples were collected for the biochemical analysis (ALT, AST, BRU, and URE). Tissue samples from various organs were collected for tissue sections. The tissue sections were analyzed by H&E staining and immunohistochemistry analysis.

##### Pharmacokinetic Study

To investigate the pharmacokinetic profile of AHP‐DRI‐12 and PEG‐tAHP‐DRI, SD rats were intravenously injected with FITC‐labeled AHP‐DRI‐12 and PEG‐tAHP‐DRI and blood samples were collected at 0.5, 1, 2, 4, 6, 24, 30, 48, 54, and 72 h. The DR6‐targeting peptide level of blood samples was measured by a microplate reader.

##### Biodistribution Study

To quantify the biodistribution, mice were intravenously injected with Cy7‐labeled peptides. Mice were sacrificed at 6, 12, and 24 h after injection. All organs were harvested and subjected to ex vivo fluorescence imaging.

##### Peptides Stability in Serum

FITC‐labeled peptides were added into 25% rat serum and incubated at 37 °C. After incubation at 0, 4, 8, 24, 48, 96 h, 100 µL solution was collected and acetonitrile (containing 0.1% TFA) was added to precipitate the protein in the serum. The serum samples were incubated at 4 °C for 20 min and then centrifuged at 12 000 rpm for 10 min. A total of 20 µL supernatant was measured by a microplate reader.

##### Statistical Analysis

Data are presented as the means ± standard deviations (SD) and analyzed by Student's *t*‐test with GraphPad Prism software 8.0. *p* < 0.05 was considered statistically significant (NS: *p* > 0.05, 0.01 < *p < 0.05, 0.001 < **p <0.01, ***p < 0.001).

## Conflict of Interest

The authors declare no conflict of interest.

## Supporting information

Supporting Information

## Data Availability

Research data are not shared.
