# Supplementary material for: Multi‐Arm PEG/Peptidomimetic Conjugate Inhibitors of DR6/APP Interaction Block Hematogenous Tumor Cell Extravasation
Source: Adv Sci (Weinh). 2021 Mar 18;8(11):2003558. doi: 10.1002/advs.202003558 (PMC8188212; doi:10.1002/advs.202003558)
Supplement: Supplementary file 1 — Supporting Information [file ADVS-8-2003558-s001.pdf]

© 2021 Wiley-VCH GmbH

ADVANCED SCIENCE
Open Access

Supporting Information

for *Adv. Sci.*, DOI: 10.1002/advs.202003558

Multi-Arm PEG/Peptidomimetic Conjugate Inhibitors of DR6/APP Interaction Block Hematogenous Tumor Cell Extravasation

Liting Wang, Qing Shen, Hongze Liao, Hao Fu, Qi Wang, Jian Yu, Wei Zhang, Chuanrong Chen, Yang Dong, Xupeng Yang, Qianqian Guo, Jiali Zhang, Jian Zhang, Wei Zhang, Houwen Lin,* and Yourong Duan**

Supporting Information

Multi-Arm PEG/Peptidomimetic Conjugate Inhibitors of DR6/APP Interaction Block Hematogenous Tumor Cell Extravasation

Liting Wang, Qing Shen, Hongze Liao, [*] *Hao Fu, Qi Wang, Jian Yu, Wei Zhang, Chuanrong Chen, Yang Dong, Xupeng Yang, Qianqian Guo, Jiali Zhang, Jian Zhang, Wei Zhang, Houwen Lin,* [*] *and Yourong Duan*[*]

Dr. L. Wang, Dr. Q. Shen, Dr. H. Fu, Dr. J. Yu, Dr. W. Zhang, Dr. C. Chen, Dr. Y. Dong, X. Yang, Dr. Q. Guo, Dr. J. Zhang, Prof. H. Lin, Prof. Y. Duan

State Key Laboratory of Oncogenes and Related Genes, Shanghai Cancer Institute, School of Biomedical Engineering, Renji Hospital, School of Medicine, Shanghai Jiao Tong University, Shanghai 200032, China.
E-mail: yrduan@shsci.org; franklin67@126.com

Dr. H. Liao, Prof. W. Zhang, Prof. H. Lin
Research Center for Marine Drugs, State Key Laboratory of Oncogenes and Related Genes, Department of Pharmacy, Renji Hospital, School of Medicine, Shanghai Jiao Tong University, Shanghai 200127, China.
E-mail: lhzseed@live.com or

Dr. Q. Wang
Shanghai Key Laboratory of Functional Materials Chemistry, School of Chemistry and Molecular Engineering, East China University of Science and Technology, Shanghai 200237, China.

Prof. J. Zhang
Department of Pathophysiology, Key Laboratory of Cell Differentiation and Apoptosis of Ministry of Education, Shanghai Jiao Tong University School of Medicine, Shanghai 200025, China.

Table S1. Amino acid sequences of twenty-four peptides and the corresponding K_D values. [a]

ID	Sequence	K_D ± SEM[b] (nM)
AHP-01	WTLSINS	1153.7 ± 7.94
AHP-02	FQFRLQS	NB[c]
AHP-03	YLPAPLP	688.5 ± 11.20
AHP-04	ANLLYES	NB
AHP-05	YYSGNNL	1096 ± 10.04
AHP-06	KLGPVCP	NB
AHP-07	YSLHASP	NB
AHP-08	HSTAPQL	NB
AHP-09	KLVPEPS	NB
AHP-10	NGSTHER	527.8 ± 12.09
AHP-11	GFSHYFP	NB
AHP-12	TIDATTP	321.8 ± 11.24
AHP-13	SHTDTNQ	1153.7 ± 7.94
AHP-14	AFMYSLP	457.3 ± 11.56
AHP-15	TIPKYER	NB
AHP-16	KFLQVTN	NB
AHP-17	HSYPYTQ	NB
AHP-18	TTYMPKM	NB
AHP-19	MPPFRHD	NB
AHP-20	LPRSPLA	NB
AHP-21	AGHFSLW	NB
AHP-22	LMTTKSD	NB
AHP-23	GGLSMTR	4458.8 ± 5.85
AHP-24	NHQRLLP	1093.5 ± 262

[a] K_D values of peptides to DR6 were assessed as described in the Materials and Methods.

[b] K_D values shown are the average of three independent experiments in triplicate.

[c] NB = No binding.

Table S2. Amino acid sequences of D-peptides and DRI-peptides and the corresponding K_D values.

ID	Sequence	K_D ± SEM (nM)
AHP-D-03	D-(YLPAPLP)	4734.0 ± 8.80
AHP-D-10	D-(NGSTHER)	27110.0 ± 47383
AHP-D-12	D-(TIDATTP)	55130.0 ± 27585
AHP-D-13	D-(SHTDTNQ)	3174 ± 8.80
AHP-DRI-03	D-(PLPAPLY)	1219 ± 7.02
AHP-DRI-10	D-(REHTSGN)	17810 ± 37081
AHP-DRI-12	D-(PTTADIT)	225.19 ± 4.30
AHP-DRI-13	D-(QNTDTHS)	862.7 ± 6.59

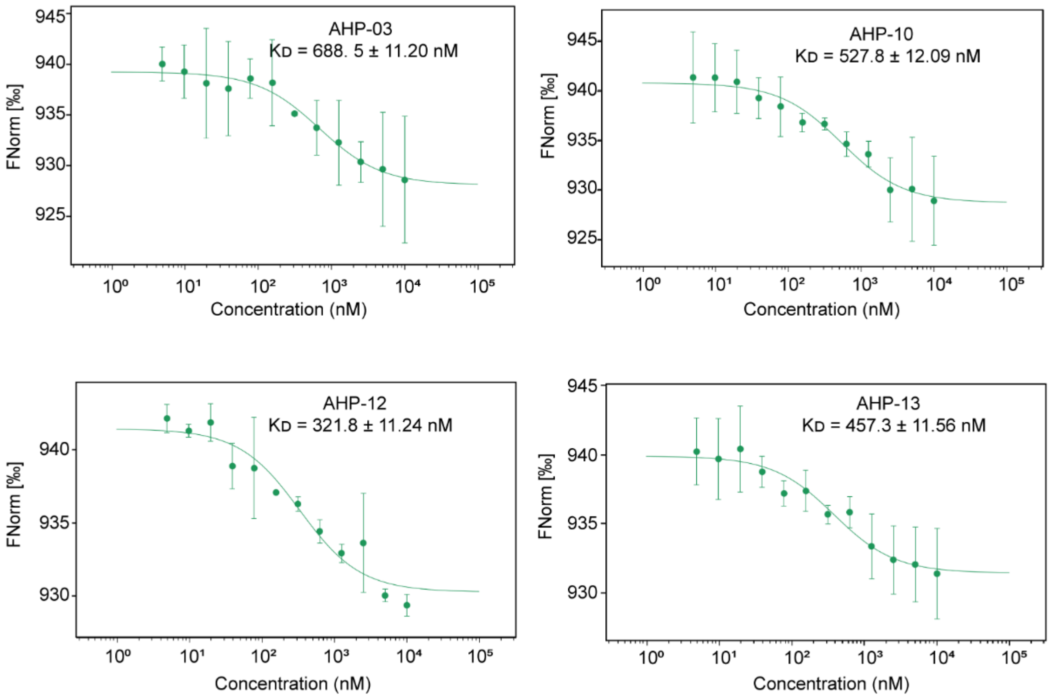

Figure S1. The binding curve of AHP-03, AHP-10, AHP-12 and AHP-13 to DR6 protein were measured by MST, respectively.

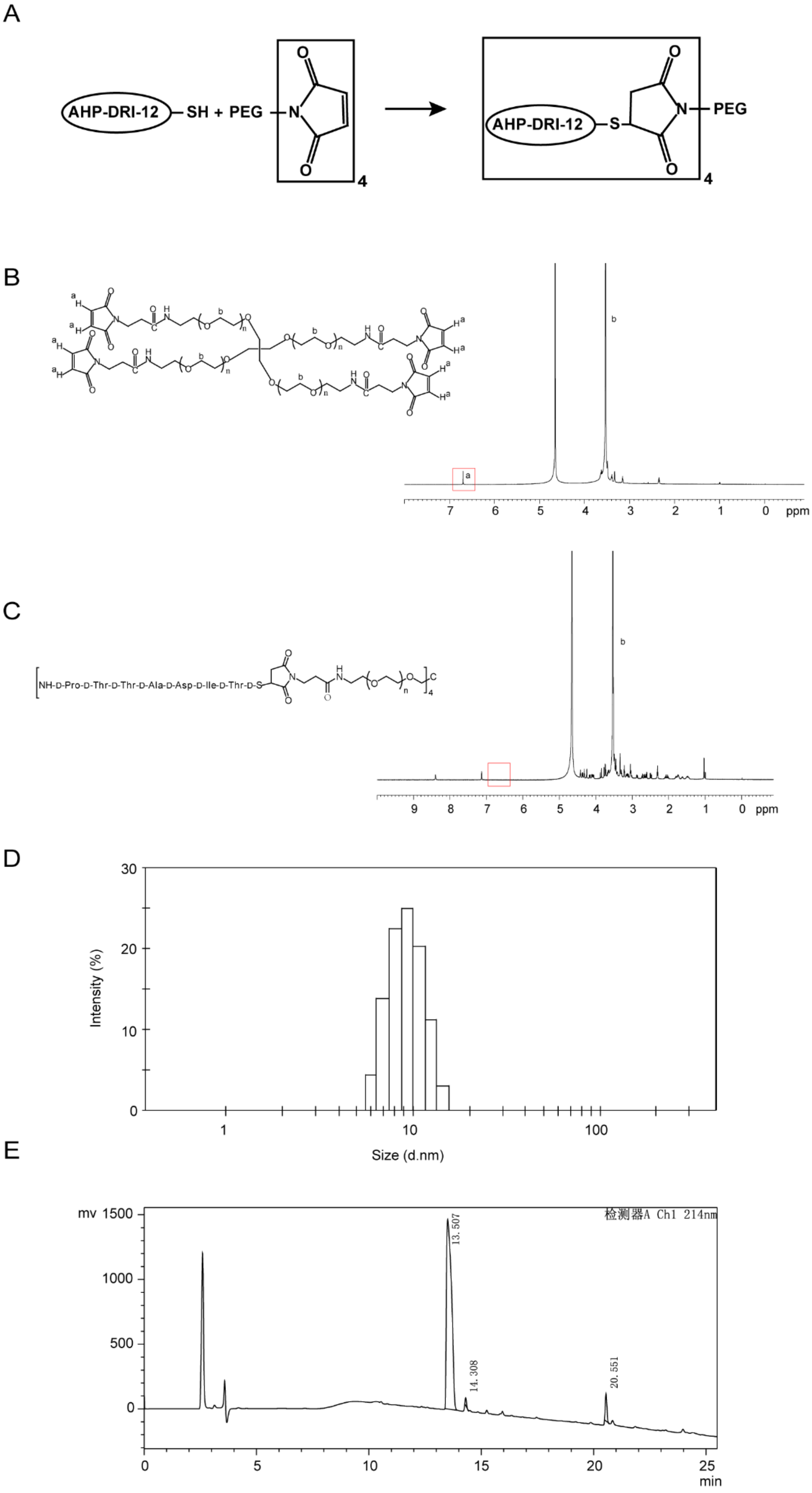

A
AHP-DRI-12
SH + PEG
N
O
4
PEG
S
B
a
b
n
ppm
C
NH-D-Pro-D-Thr-D-Thr-D-Ala-D-Asp-D-Ile-D-Thr-D-S
D
Intensity (%)
Size (d.nm)
E
mv
检测器A Ch1 214nm
13.507
14.308
20.551
min

Figure S2. Synthesis of PEG-tAHP-DRI. A) Schematic presentation of PEG-tAHP-DRI. **B)** ^{1}H NMR spectra of 4-arm-PEG$_{5k}$ in D_2O. **C)** ^{1}H NMR spectra of PEG-tAHP-DRI in D_2O. **D)** Size distribution of PEG-tAHP-DRI. **E)** HPLC curve of PEG-tAHP-DRI.

A

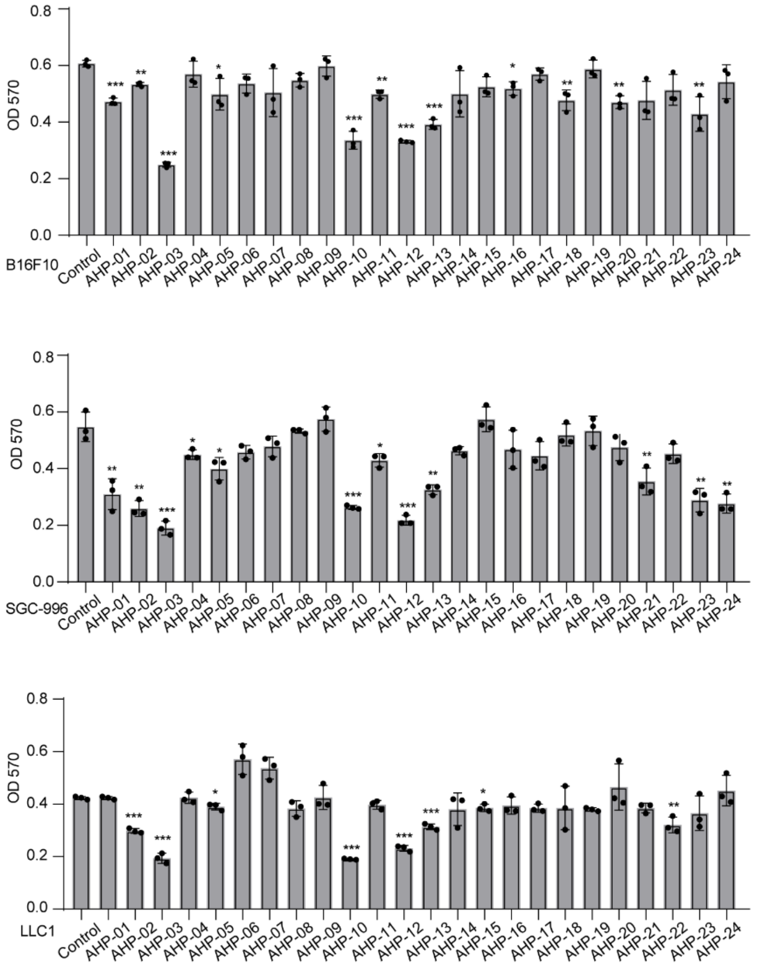
OD 570
0.0
0.2
0.4
0.6
0.8
B16F10
SGC-996
LLC1
Control
AHP-01
AHP-02
AHP-03
AHP-04
AHP-05
AHP-06
AHP-07
AHP-08
AHP-09
AHP-10
AHP-11
AHP-12
AHP-13
AHP-14
AHP-15
AHP-16
AHP-17
AHP-18
AHP-19
AHP-20
AHP-21
AHP-22
AHP-23
AHP-24

B

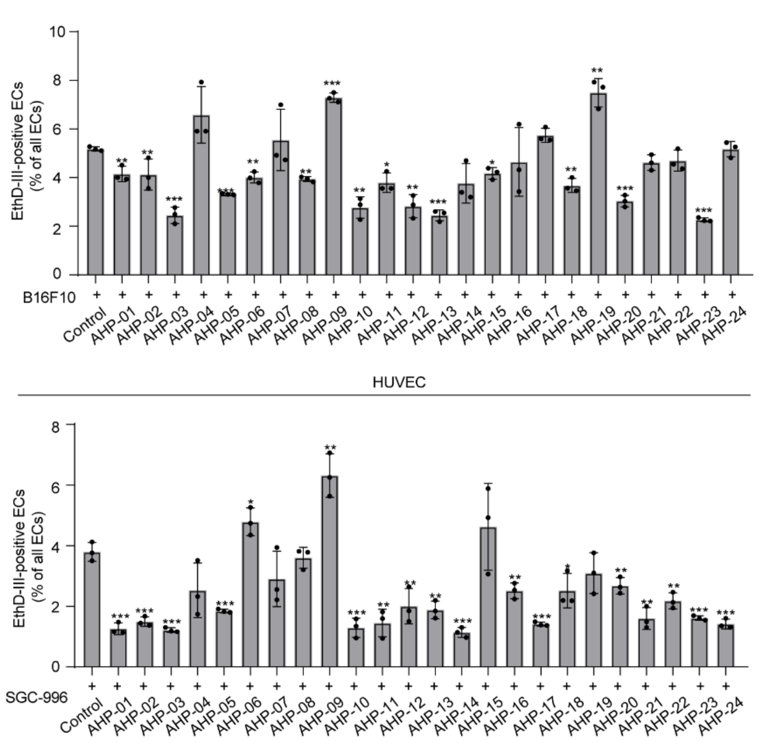
HUVEC
EthD-III-positive ECs
(% of all ECs)
0
2
4
6
8
10
B16F10
SGC-996
Control
AHP-01
AHP-02
AHP-03
AHP-04
AHP-05
AHP-06
AHP-07
AHP-08
AHP-09
AHP-10
AHP-11
AHP-12
AHP-13
AHP-14
AHP-15
AHP-16
AHP-17
AHP-18
AHP-19
AHP-20
AHP-21
AHP-22
AHP-23
AHP-24

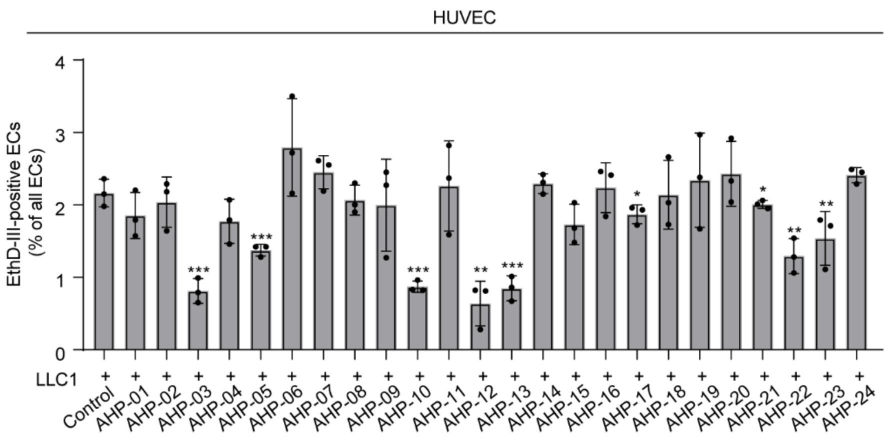

Figure S3. Screening of twenty-four DR6-targeting peptides. A) Cell attachment of different TCs to culture plate coated with DR6 protein after treated twenty-four peptides. **B)** HUVECs necroptosis when co-cultured with different TCs after treated with twenty-four peptides. Data are means ± SD (n = 3), and analyzed with GraphPad Prism 8.0. *P < 0.05, **P < 0.01, ***P < 0.005 calculated by unpaired two-tailed Student's t-test. NS indicates P > 0.05.

A

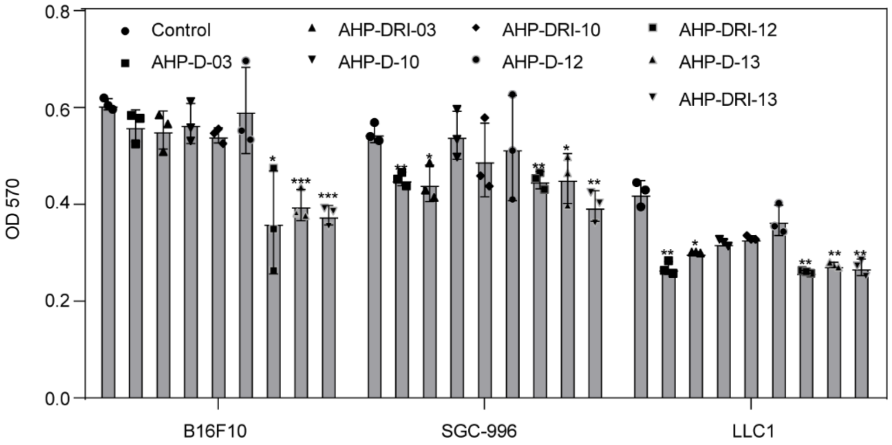
Control
AHP-D-03
AHP-DRI-03
AHP-D-10
AHP-DRI-10
AHP-D-12
AHP-DRI-12
AHP-D-13
AHP-DRI-13
OD 570
0.0
0.2
0.4
0.6
0.8
B16F10
SGC-996
LLC1

B

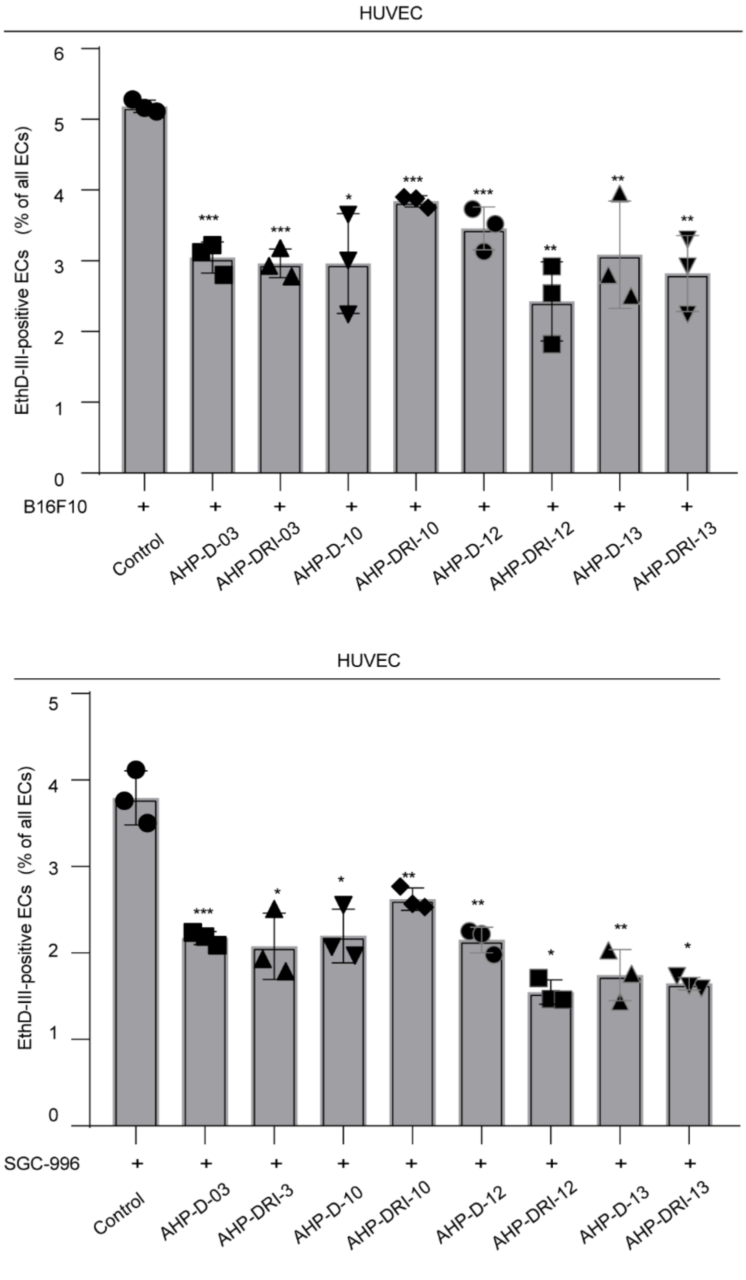
HUVEC
EthD-III-positive ECs (% of all ECs)
B16F10
Control
AHP-D-03
AHP-DRI-03
AHP-D-10
AHP-DRI-10
AHP-D-12
AHP-DRI-12
AHP-D-13
AHP-DRI-13
HUVEC
EthD-III-positive ECs (% of all ECs)
SGC-996
Control
AHP-D-03
AHP-DRI-3
AHP-D-10
AHP-DRI-10
AHP-D-12
AHP-DRI-12
AHP-D-13
AHP-DRI-13

C

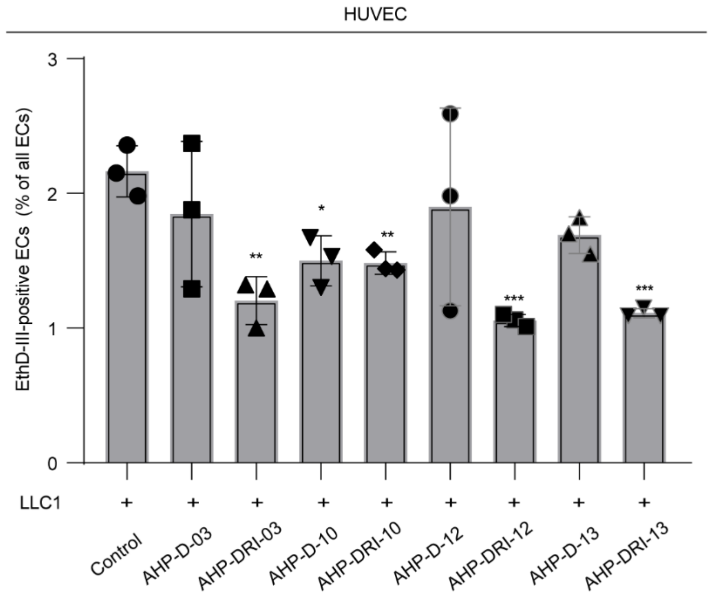

Figure S4. Screening of D-peptides and DRI-peptides. A) Cell attachment of different TCs to culture plate coated with DR6 protein after treated with eight peptides. **B)** HUVECs necroptosis when co-cultured with different TCs after treated with eight peptides. Data are means ± SD (n = 3), and analyzed with GraphPad Prism 8.0. *P < 0.05, **P < 0.01, ***P < 0.005 calculated by unpaired two-tailed Student's t-test. NS indicates P > 0.05.

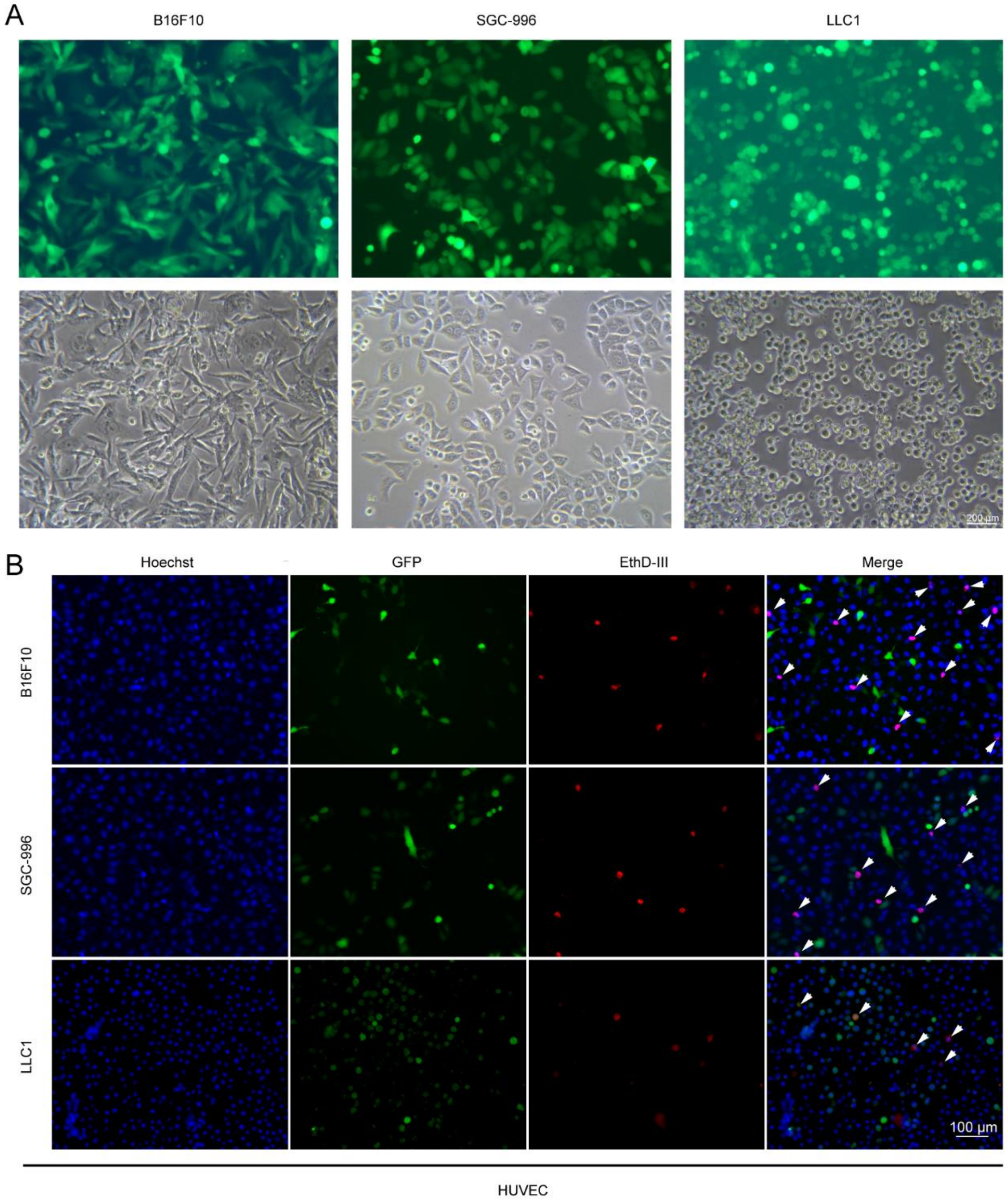

Figure S5. A) B16F10, SGC-996, LLC1 cells stably expressed GFP. **B) F**luorescent images of HUVECs cultured in the presence of GFP-TCs (green) and stained as indicated. Arrowheads, EthD-III-positive HUVECs (red).

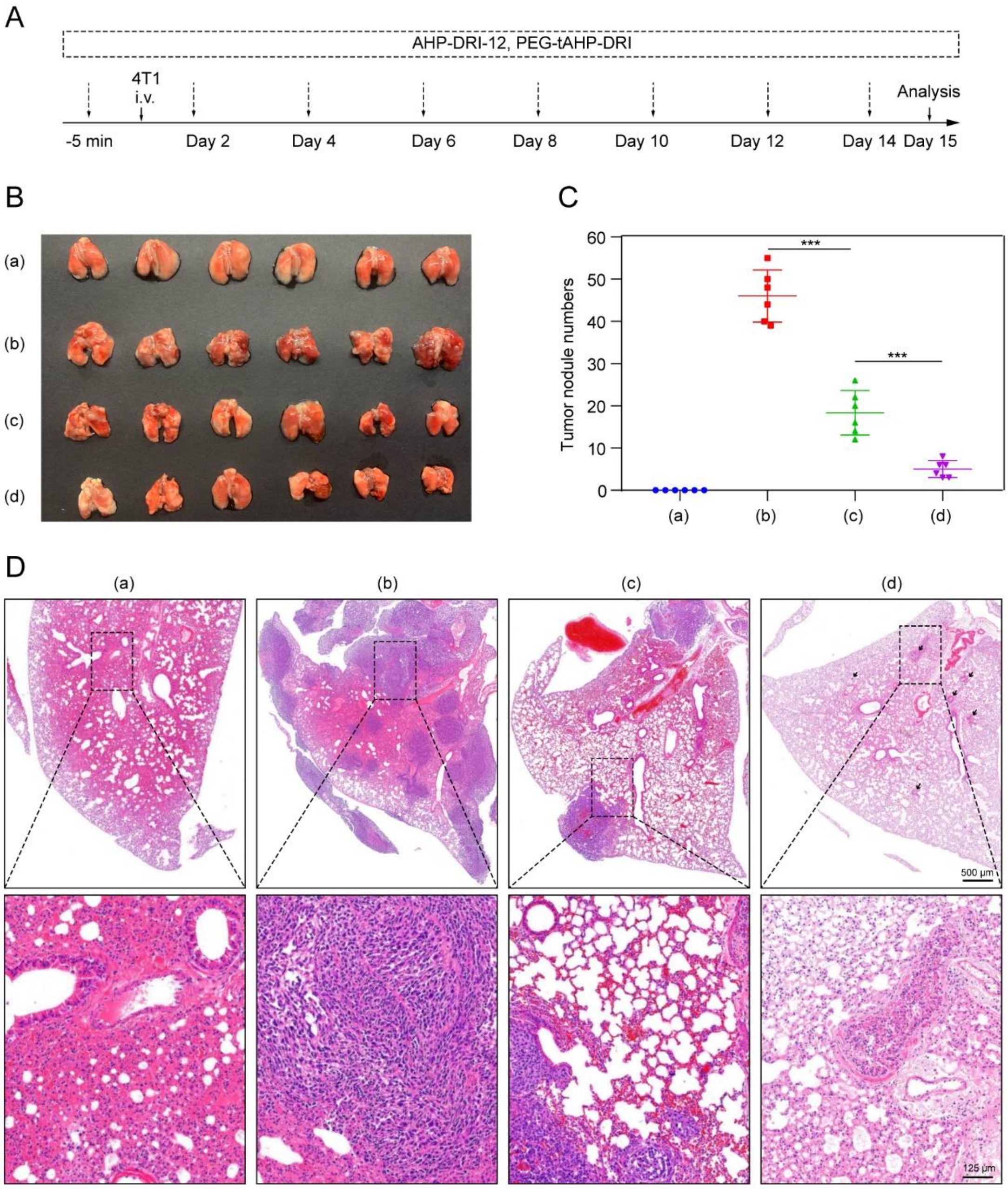

Figure S6. A) Experimental design. **B**) Photographs of the lungs 15 days after i.v. injection of 4T1. (a) saline, (b) 4T1 + saline (vehicle), (c) 4T1 + AHP-DRI-12, (d) 4T1 + PEG-tAHP-DRI. **C**) Metastasis nodules of lungs were quantified. **D**) H&E staining of lung sections. Data are means ± SD (n = 6), and analyzed with GraphPad Prism 8.0. *P < 0.05, **P < 0.01, ***P < 0.005 calculated by unpaired two-tailed Student's t-test. NS indicates P > 0.05.

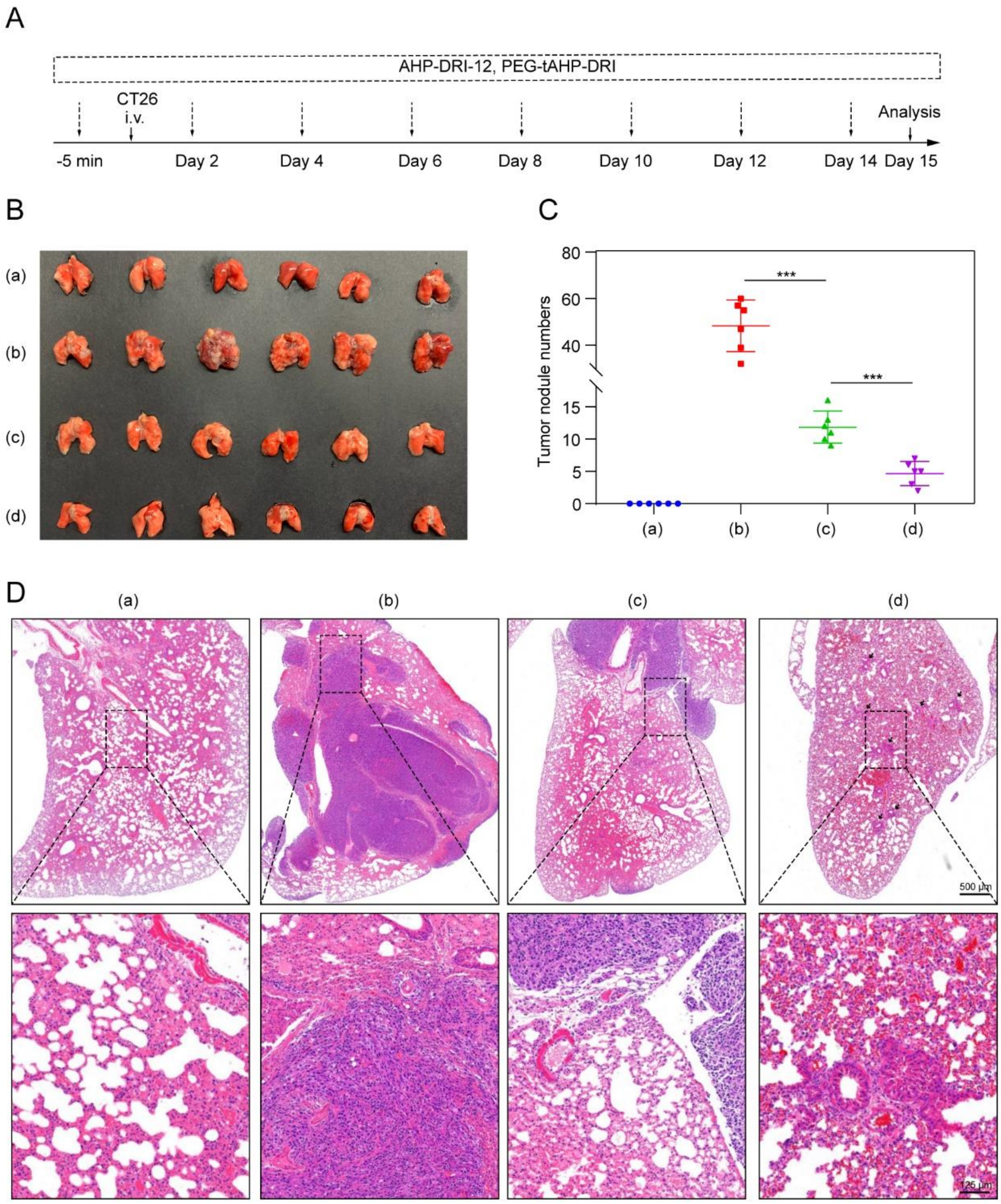

Figure S7. A) Experimental design. **B)** Photographs of the lungs 15 days after i.v. injection of CT26. (a) saline, (b) CT26 + saline (vehicle), (c) CT26 + AHP-DRI-12, (d) CT26 + PEG-tAHP-DRI. **C)** Metastasis nodules of lungs were quantified. **D)** H&E staining of lung sections. Data are means ± SD (n = 6), and analyzed with GraphPad Prism 8.0. *P < 0.05, **P < 0.01, ***P < 0.005 calculated by unpaired two-tailed Student's t-test. NS indicates P > 0.05.

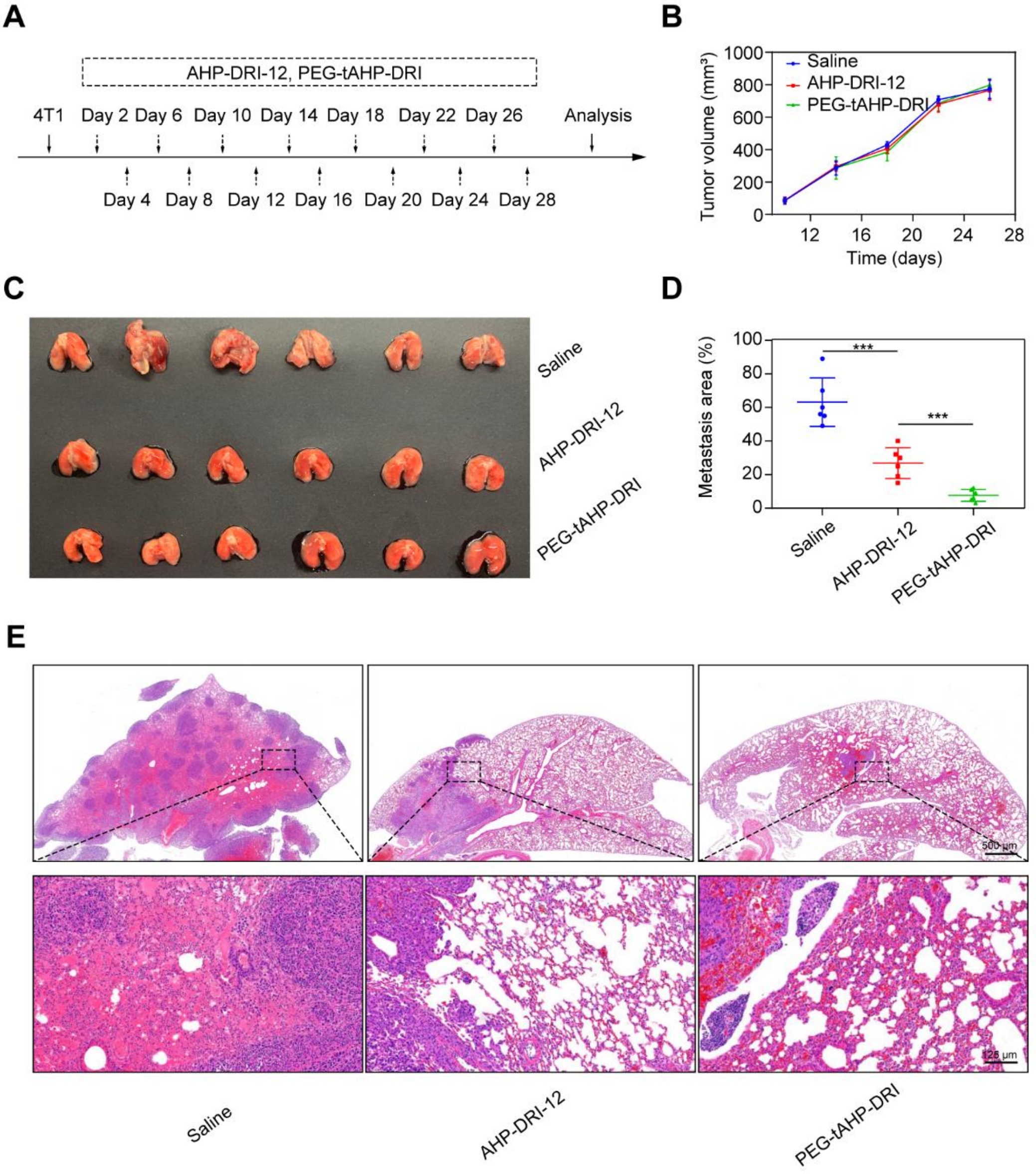

Figure S8. A) Experimental design. **B)** Orthotopic tumor growth of 4T1. **C)** Photographs of the lungs 30 days after 4T1 injection. **D)** Metastasis areas of lungs were quantified. **E)** H&E staining of lung sections. Data are means ± SD (n = 6), and analyzed with GraphPad Prism 8.0. *P < 0.05, **P < 0.01, ***P < 0.005 calculated by unpaired two-tailed Student's t-test. NS indicates P > 0.05.

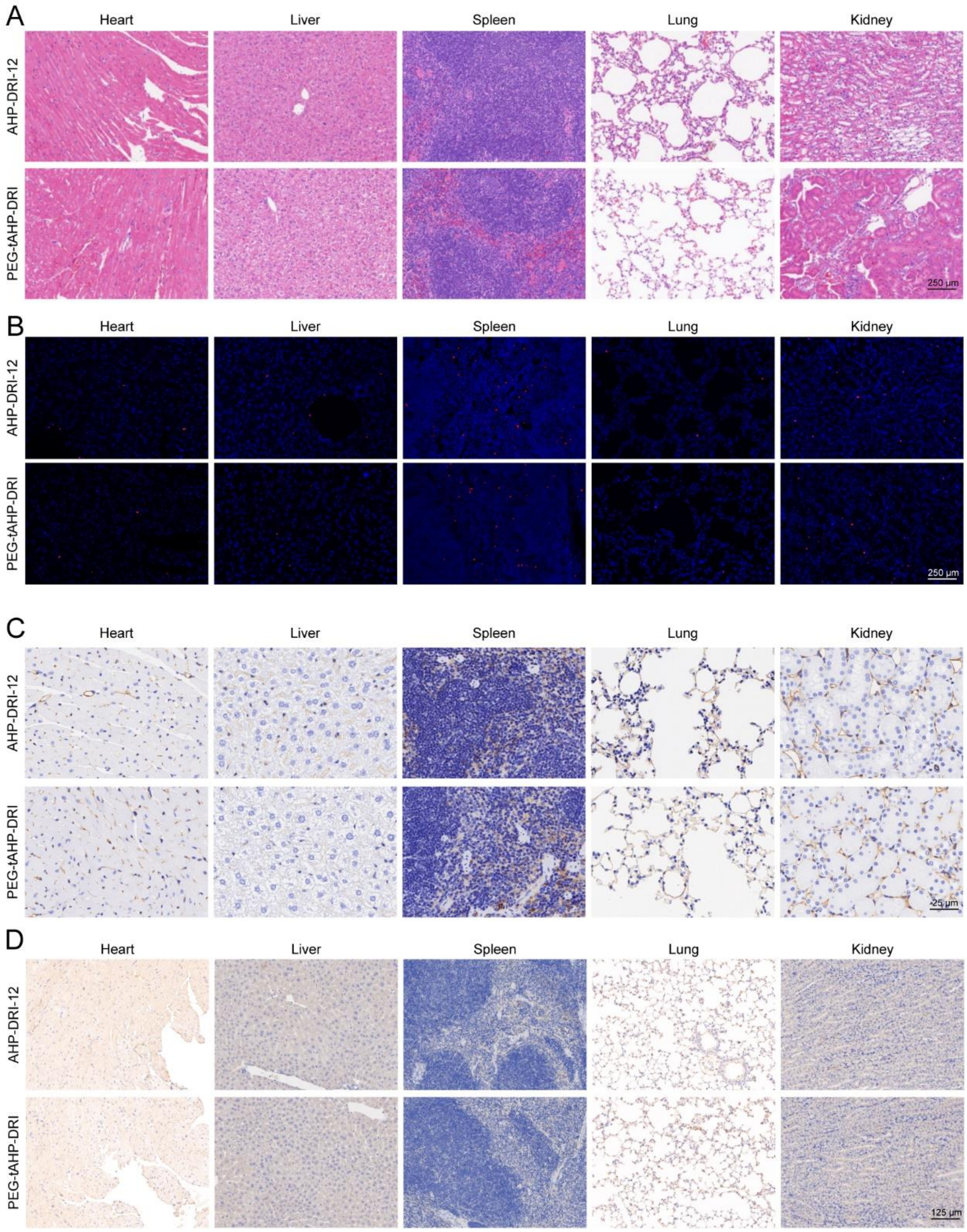
A
Heart
Liver
Spleen
Lung
Kidney
AHP-DRI-12
PEG-tAHP-DRI
250 μm
B
Heart
Liver
Spleen
Lung
Kidney
AHP-DRI-12
PEG-tAHP-DRI
250 μm
C
Heart
Liver
Spleen
Lung
Kidney
AHP-DRI-12
PEG-tAHP-DRI
25 μm
D
Heart
Liver
Spleen
Lung
Kidney
AHP-DRI-12
PEG-tAHP-DRI
125 μm

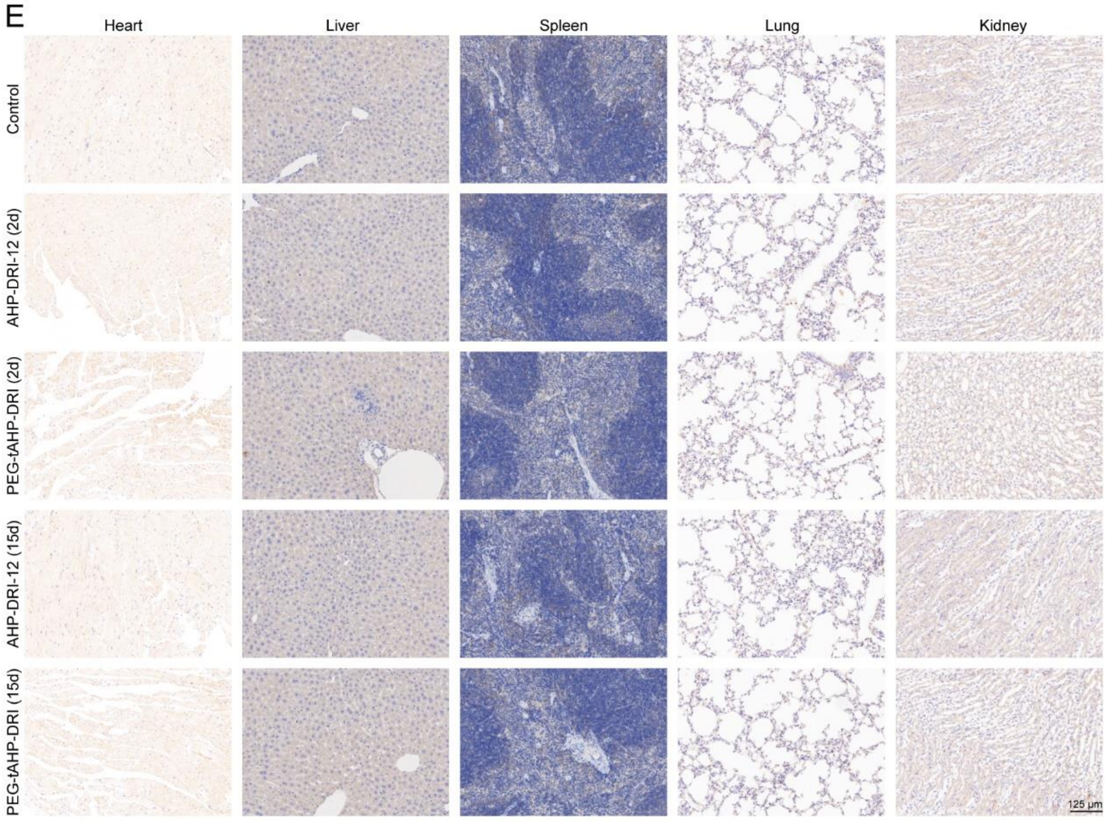

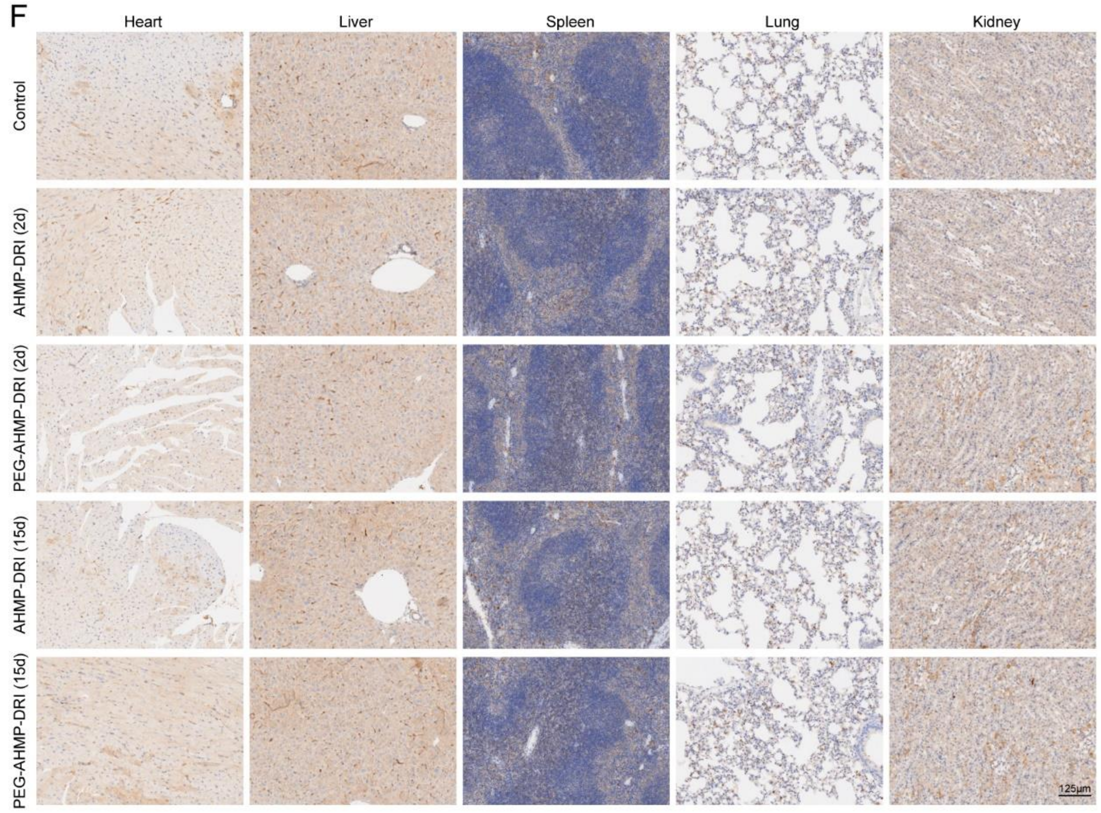

Figure S9. A, **B)** Tissue sections (heart, liver, spleen, lung and kidney) of healthy mice 2 days after injected with AHP-DRI-12 or PEG-tAHP-DRI were stained with H&E (**A**) and Tunel (**B**). **C, D)** Tissue

sections (heart, liver, spleen, lung and kidney) of healthy mice 2 days after injected with AHP-DRI-12 or PEG-tAHP-DRI were stained with CD31 **(C)** and p-RIPK1 **(D)**. **E**, **F)** Tissue sections (heart, liver, spleen, lung and kidney) of healthy mice 2 days and 15 days after injected with AHP-DRI-12 or PEG-tAHP-DRI were stained with p-IκB-α (**E**) and caspase 8 (**F**).

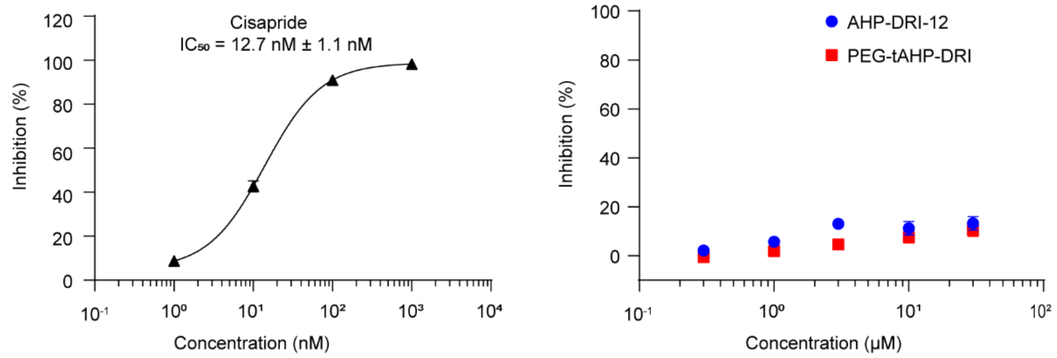

Figure S10. Current change of hERG induced by cisapride, AHP-DRI-12 or PEG-tAHP-DRI.

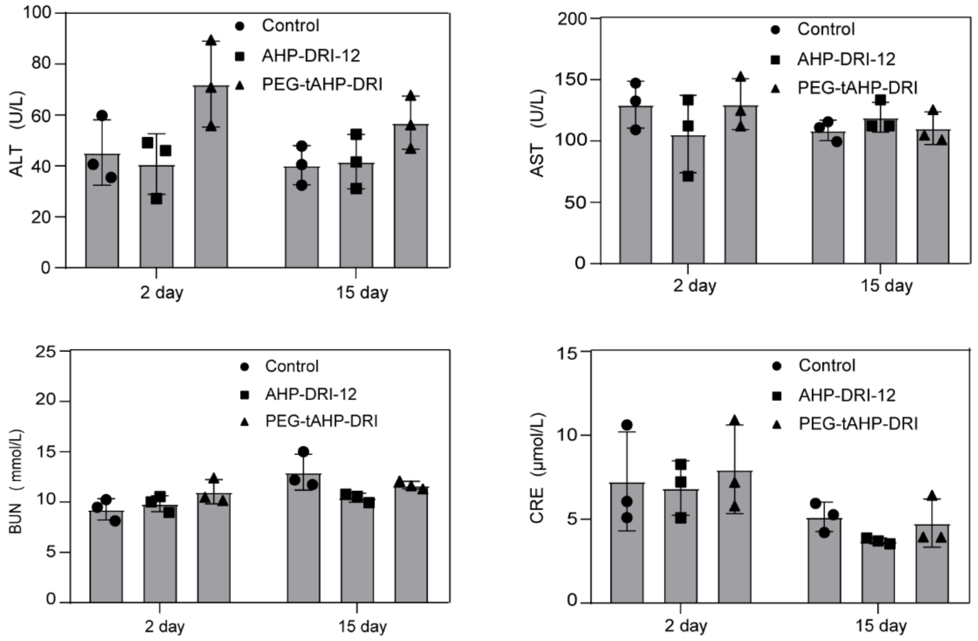

Figure S11. Serum ALT, AST, BUN and CRE levels after i.v. injection of AHP-DRI-12 and PEG-tAHP-DRI. Data are means ± SD (n = 3), and analyzed with GraphPad Prism 8.0. *P < 0.05, **P < 0.01, ***P < 0.005 calculated by unpaired two-tailed Student's t-test. NS indicates P > 0.05.

AHP-01: WTLSINS

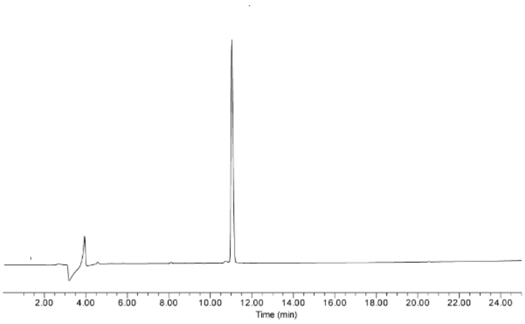

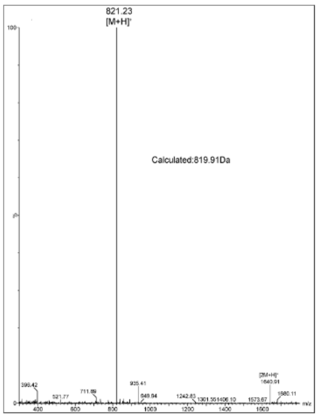

AHP-02: FQFRLQS

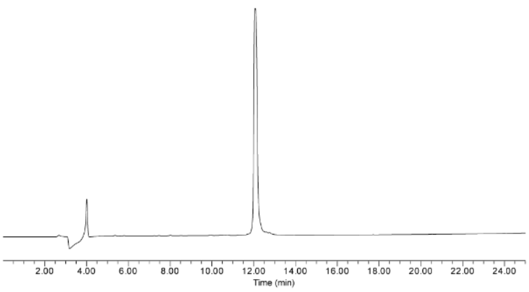

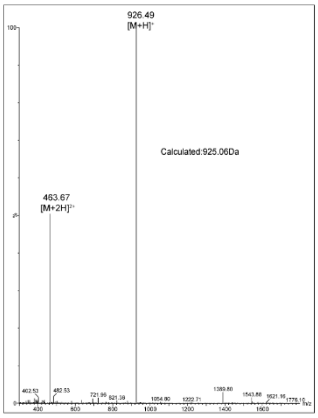

AHP-03: YLPAPLP

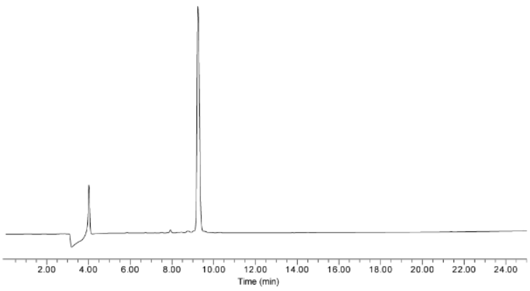

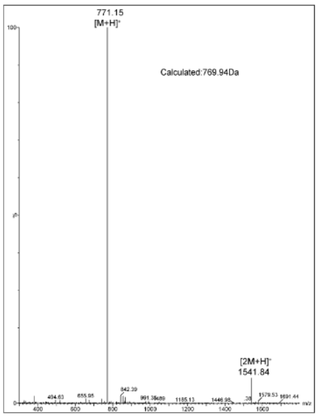

AHP-04: ANLLYES

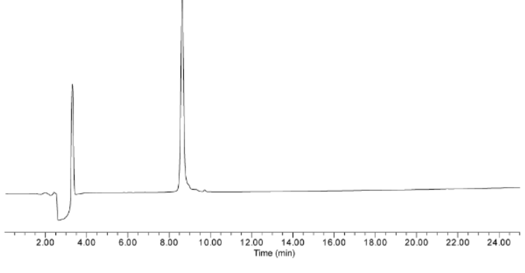

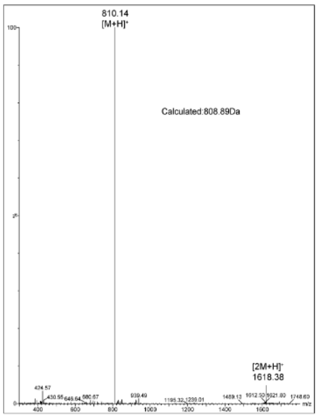

AHP-05: YYSGNNL

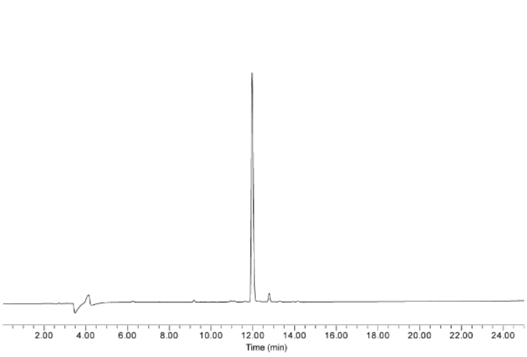

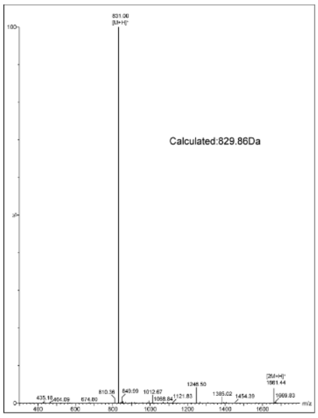

AHP-06: KLGPVCP

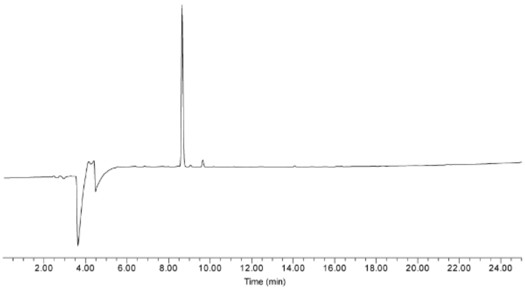

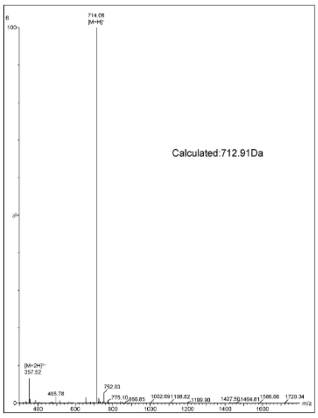

AHP-07: YSLHASP

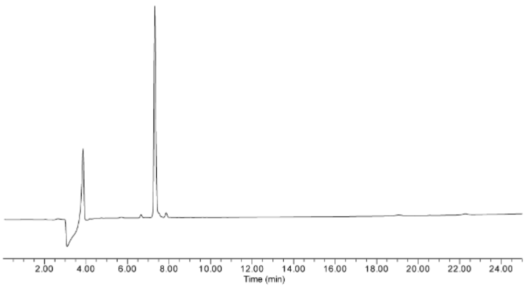

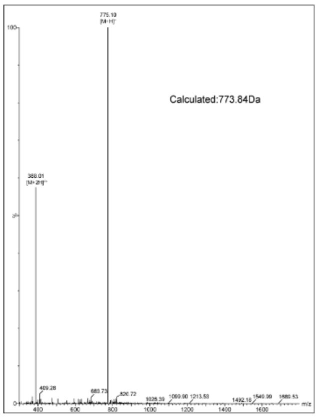

AHP-08: HSTAPQL

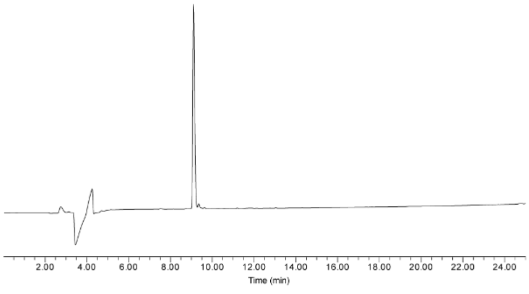

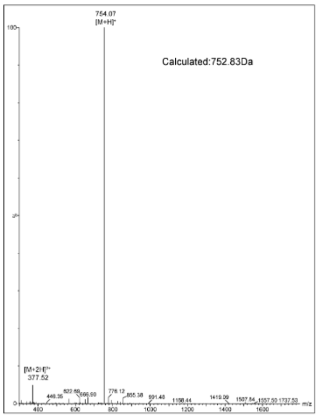

AHP-09: KLVPEPS

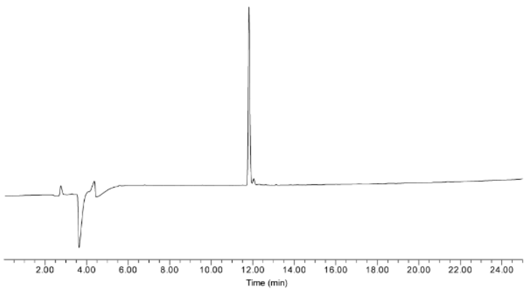

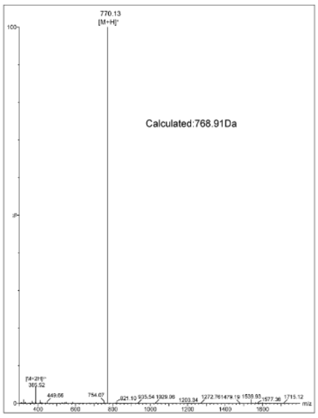

AHP-10: NGSTHER

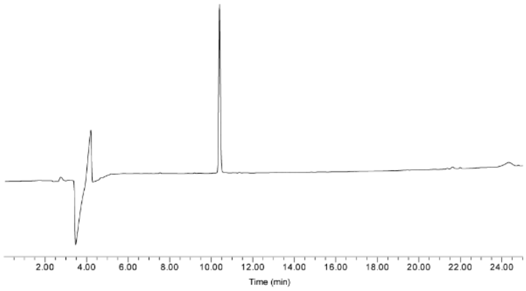

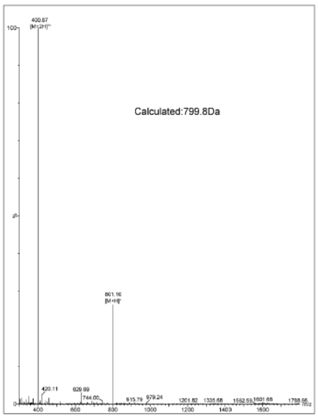

AHP-11: GFSHYFP

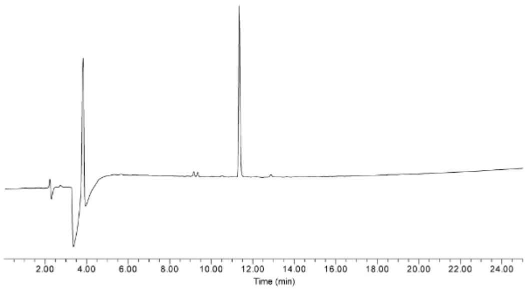

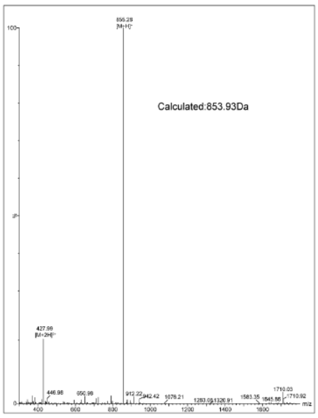

AHP-12: TIDATTP

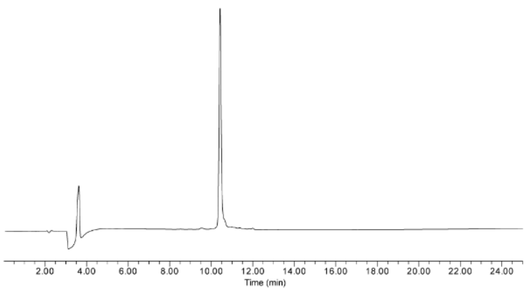

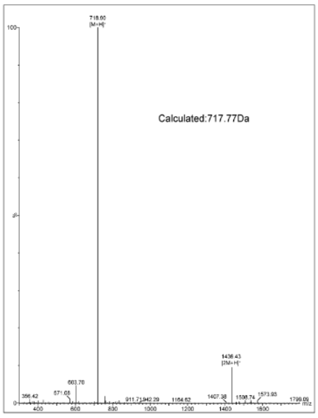

AHP-13: SHTDTNQ

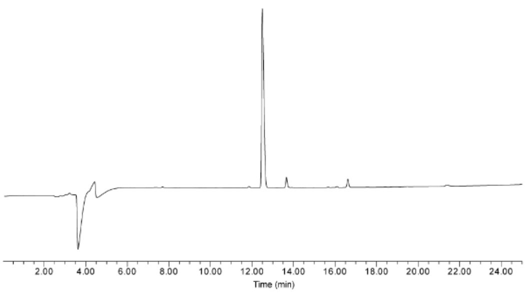

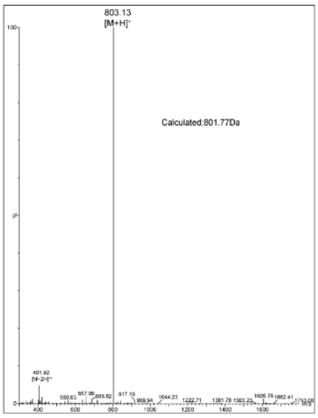

AHP-14: AFMYSLP

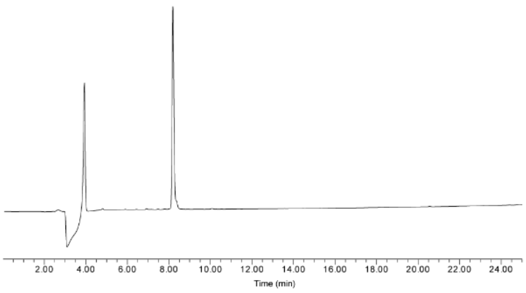

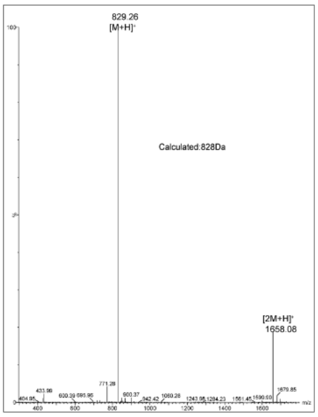

AHP-15: TIPKYER

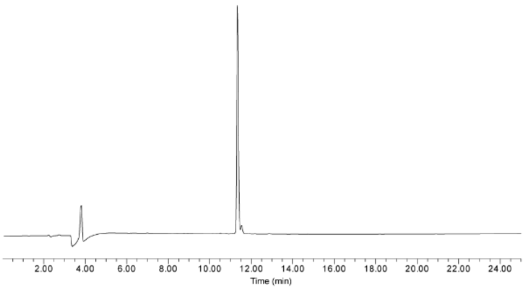

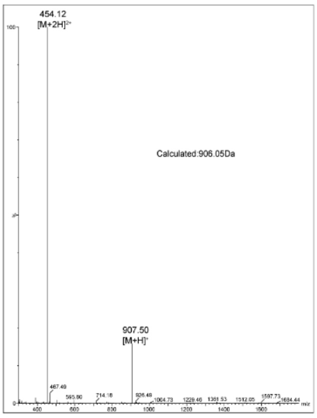

AHP-16: KFLQVTN

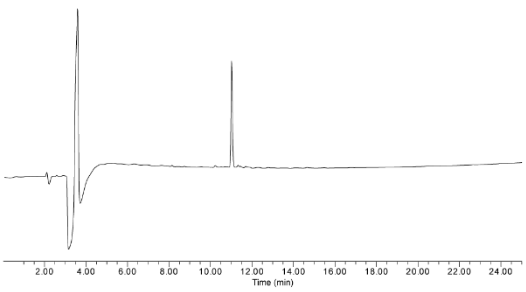

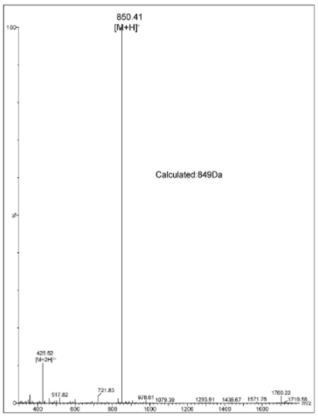

AHP-17: HSYPYTQ

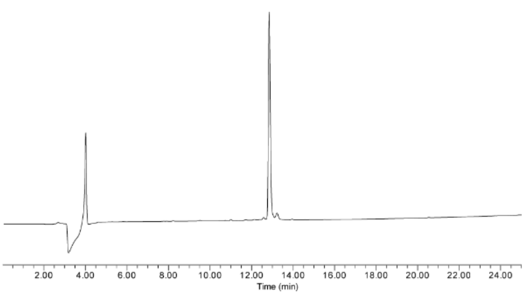

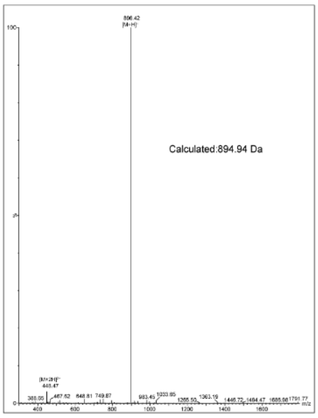

AHP-18: TTYMPKM

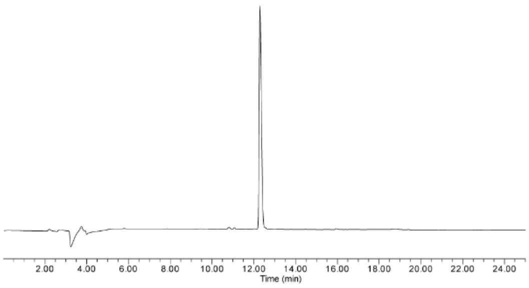

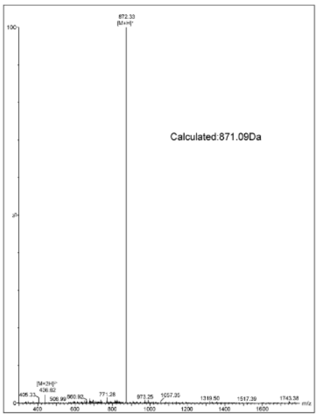

AHP-19: MPPFRHD

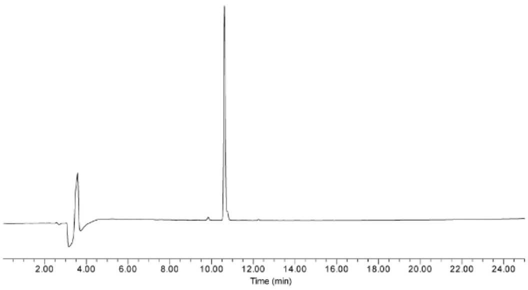

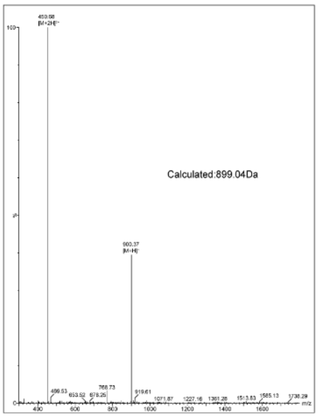

AHP20: LPRSPLA

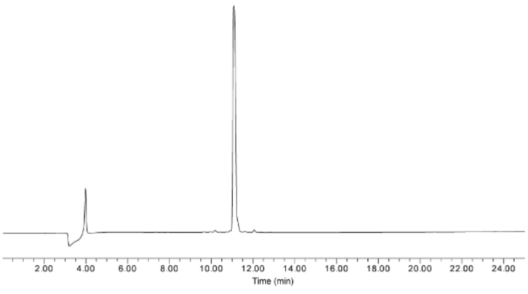

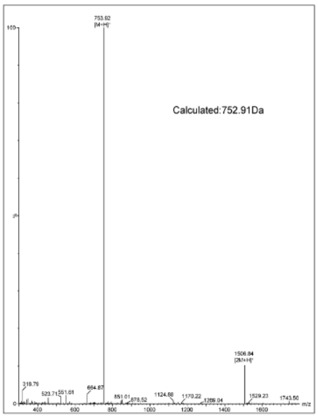

AHP-21: AGHFSLW

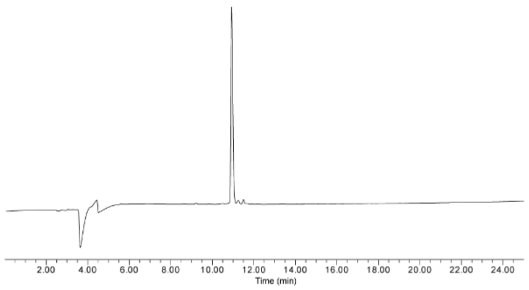

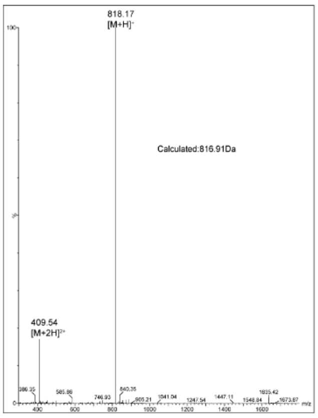

AHP-22: LMTTKSD

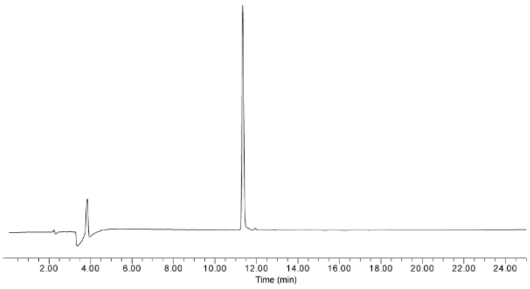

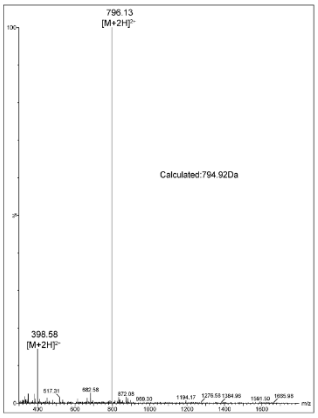

AHP-23: GGLSMTR

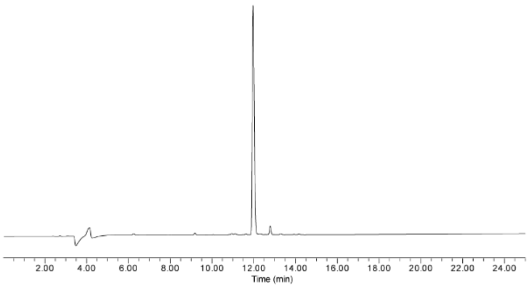

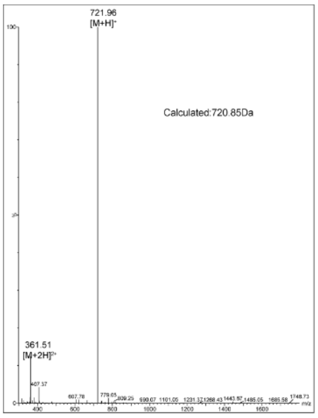

AHP-24: NHQRLLP

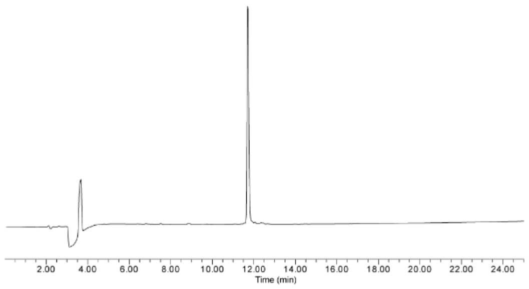

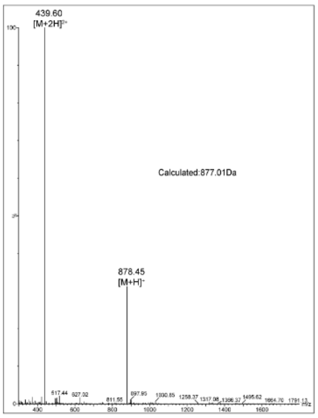

Figure S12. Characterization of twenty-four DR6-targeting peptides by HPLC (λ = 220 nm) and ESI-MS.

AHP-D-03: D-(YLPAPLP)

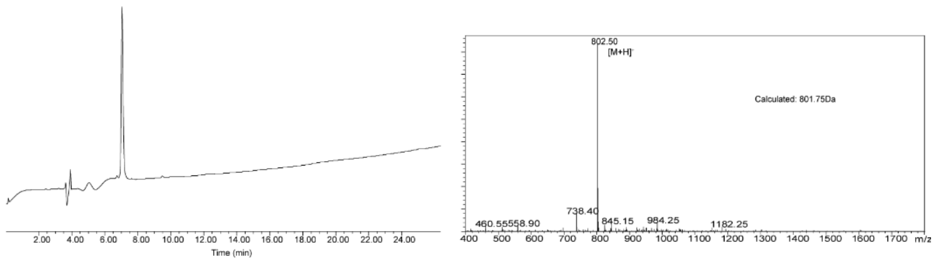

AHP-D-10: D-(NGSTHER)

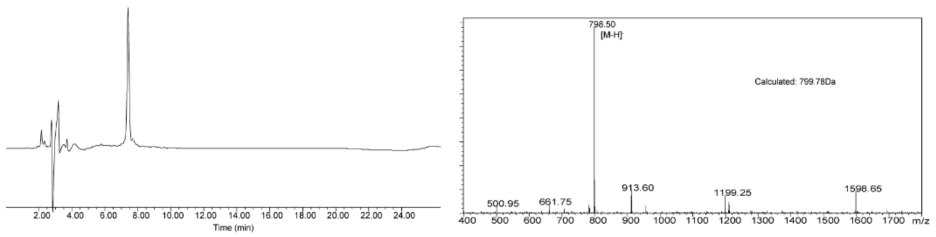

AHP-D-12: D-(TIDATTP)

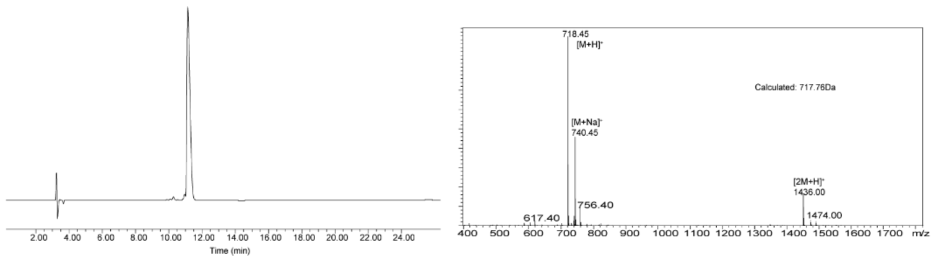

AHP-D-13: D-(SHTDTNQ)

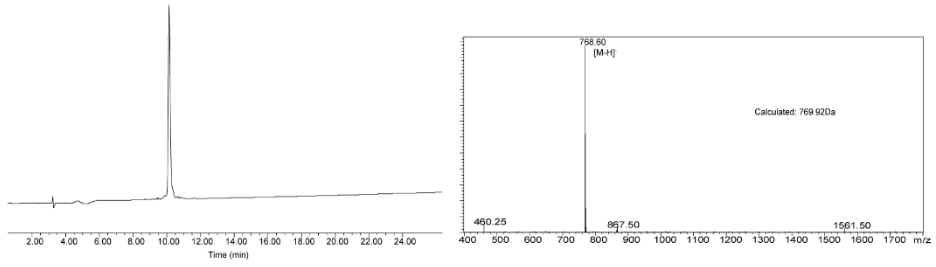

AHP-DRI-03: D-(PLPAPLY)

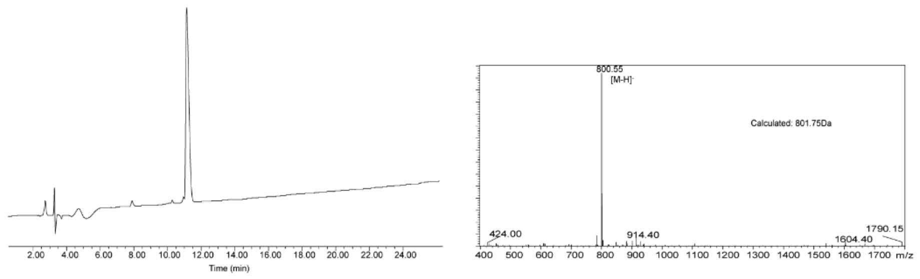

AHP-DRI-10: D-(REHTSGN)

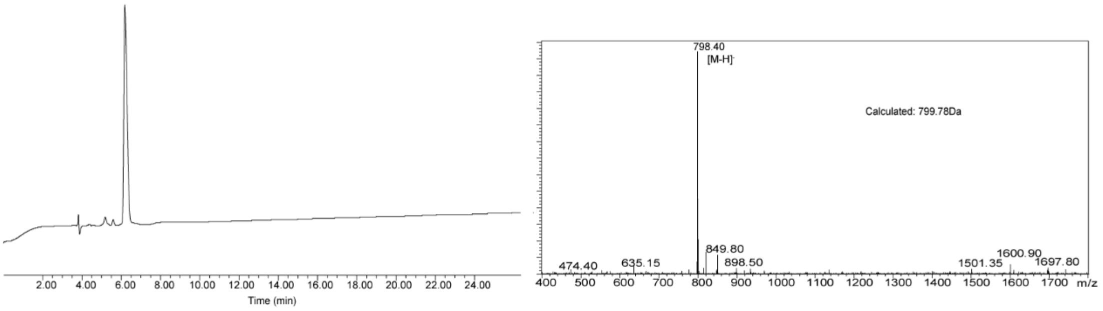

AHP-DRI-12: D-(PTTADIT)

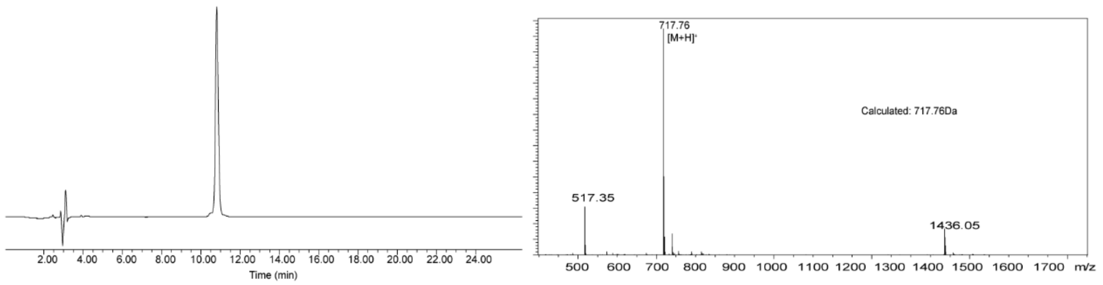

AHP-DRI-13: D-(QNTDTHS)

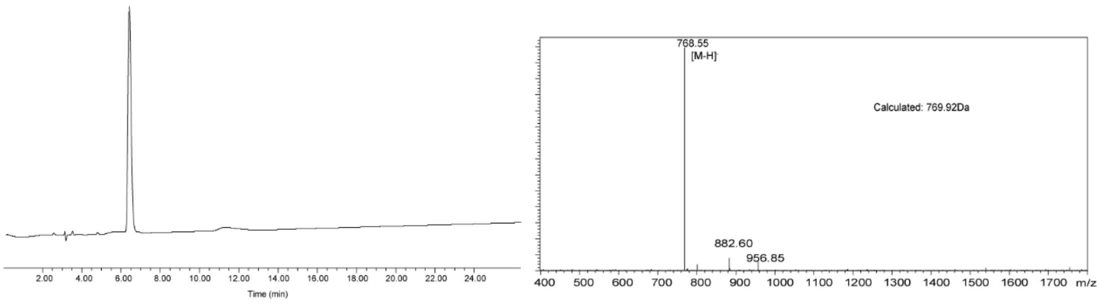

Figure S13. Characterization of D-peptides and DRI-peptides (totally composed of D amino acids) by HPLC (λ = 220 nm) and ESI-MS.
